# A Theoretical Analysis of the Geography of Schistosomiasis in Burkina Faso Highlights the Roles of Human Mobility and Water Resources Development in Disease Transmission

**DOI:** 10.1371/journal.pntd.0004127

**Published:** 2015-10-29

**Authors:** Javier Perez-Saez, Lorenzo Mari, Enrico Bertuzzo, Renato Casagrandi, Susanne H. Sokolow, Giulio A. De Leo, Theophile Mande, Natalie Ceperley, Jean-Marc Froehlich, Mariam Sou, Harouna Karambiri, Hamma Yacouba, Amadou Maiga, Marino Gatto, Andrea Rinaldo

**Affiliations:** 1 Laboratory of Ecohydrology, Ecole Polytechnique Fédérale de Lausanne (EPFL), Lausanne, Switzerland; 2 Dipartimento di Elettronica, Informazione e Bioingegneria, Politecnico di Milano, Milano, Italy; 3 Hopkins Marine Station, Stanford University, Pacific Grove, California, United States of America; 4 Marine Science Institute, University of California Santa Barbara, California, United States of America; 5 Woods Institute for the Environment, Stanford University, California, United States of America; 6 Institute International d’Ingénierie de l’Eau et de l’Environment, Ouagadougou, Burkina Faso; 7 Dipartimento ICEA, Università di Padova, Padova, Italy; University of Florida, UNITED STATES

## Abstract

We study the geography of schistosomiasis across Burkina Faso by means of a spatially explicit model of water-based disease dynamics. The model quantitatively addresses the geographic stratification of disease burden in a novel framework by explicitly accounting for drivers and controls of the disease, including spatial information on the distributions of population and infrastructure, jointly with a general description of human mobility and climatic/ecological drivers. Spatial patterns of disease are analysed by the extraction and the mapping of suitable eigenvectors of the Jacobian matrix subsuming the stability of the disease-free equilibrium. The relevance of the work lies in the novel mapping of disease burden, a byproduct of the parametrization induced by regional upscaling, by model-guided field validations and in the predictive scenarios allowed by exploiting the range of possible parameters and processes. Human mobility is found to be a primary control at regional scales both for pathogen invasion success and the overall distribution of disease burden. The effects of water resources development highlighted by systematic reviews are accounted for by the average distances of human settlements from water bodies that are habitats for the parasite’s intermediate host. Our results confirm the empirical findings about the role of water resources development on disease spread into regions previously nearly disease-free also by inspection of empirical prevalence patterns. We conclude that while the model still needs refinements based on field and epidemiological evidence, the proposed framework provides a powerful tool for large-scale public health planning and schistosomiasis management.

## Author Summary

Dynamical models of schistosomiasis infections, even spatially explicit ones, have so far only addressed spatial scales encompassing at best a few villages and the disease transmission impacts of related short-range human mobility. Here, we build from existing models of disease dynamics and spread, including a proxy of the ecology of the intermediate host of the parasite, and from generalized reproduction numbers of SIR-type systems developed for epidemics of waterborne disease, to set up large-scale projections of spatial patterns of the disease at whole country level. We ground our study in Burkina Faso in sub-Saharan Africa, and its model of social and economic development including the infrastructure built to exploit water resources, especially irrigation schemes, which have been empirically linked to enhanced disease burden. We make extensive use of remotely sensed and field data, and capitalize on ecohydrological insight. We suggest that reliable nationwide patterns of disease burden can be projected in relation to the key roles of human mobility and water resources development subsuming exposure, and claim that the case at hand provides an insightful example towards the integration of development and environmental thinking not confined to ad-hoc indicators of human development.

## Introduction

National programs for schistosomiasis control and elimination require appraising spatial patterns of endemic disease under variable conditions accounting for changing epidemiological drivers and controls inclusive of varying exposure rates, human mobility, habitat ranges for the intermediate host and the complexities of the parasite’s life cycle. Patterns of waterborne disease are unique in their spatial complexity which arise from pathogen reproduction, transport and transmission through waterways and human mobility networks, and for the corresponding challenges to morbidity and transmission control. Indeed both micro- and macro-parasitic waterborne diseases are conditioned by spatially varying natural (environmental or climatic [1–3]) and anthropogenic factors (water resources, [4–6] habitat availability and suitability [7], pathogen dispersal by river networks [8–11], and human mobility [12–16]. Here we focus on the transmission cycle of schistosomiasis, a parasitic disease, which is emblematic of the interplay among spatially varying drivers and controls. Schistosomiasis, or bilharzia, is a chronic debilitating disease caused by parasitic worms of genus *Schistosoma* that affected an estimated 249 million people around the world in 2012. A crushing 93% of these people live in Sub-Saharian Africa [17], where both the urinary and intestinal forms of the disease, caused by *S. haematobium* and *S. mansoni* respectively, are present. This figure has grown from 77% in 2006 [18]. Both forms of schistosomiasis have been reported in Burkina Faso since the early fifties, with measured prevalences prior to the implementation of Mass Drug Administration Campaigns (MDAs) within the Schistosomiasis Control Initiative (SCI) [19] systematically higher than 30% [20, 21]. A North-to-South decreasing gradient was observed for the urinary form of the disease and an opposite trend for the intestinal one [21]. The MDAs had a important impact on prevalence with immediate post-MDA prevalence levels ten times lower than pre-treatment baseline, but levels of infection have in some cases risen again in recent years, with some villages back to pre-treatment conditions [22]. Challenges to the successful control of the disease are manifold due to the complexity of the transmission cycle, which requires freshwater aquatic snails (*Bulinus spp*. or *Biomphalaria spp*. for *S. haematobium* and *S. mansoni* respectively) as obligate intermediate hosts. The transmission cycle consists of the excretion of parasite eggs from human to water bodies where they hatch into miracidia, the first larval stage, which infect the aquatic snail intermediate host. Asexual reproduction therein produces second-stage larvae called cercariae which infect humans through skin penetration. Once in the human host, they migrate in the system, mature into adult schistosomes and mate in the capillary surrounding the bladder or the intestine depending on the parasite genus leading to egg production and excretion. Environmental, climatic, ecological and socio-economic factors drive the transmission cycle by conditioning both snail and human probability of infection [12]. Dynamical models of schistosomiasis [23, 24], and their spatial extension to connected environments [12], provide the opportunity to show the applicability of a general mathematical framework for analysing the disease invasion conditions and the resulting spatial patterns of human schistosomiasis which could inform control and elimination programs. With this goal, we study schistosomiasis spread and persistence in the context of Burkina Faso with an emphasis on the roles of human mobility and water resources development.

The biological pace imposed by the pathogen’s life cycle makes the role of the obligate intermediate host key in disease spread on longer time scales than micro-parasitic waterborne diseases such as epidemic cholera, typically demonstrating outbreak waves [9, 16] and showing forest fire-like revamping behaviour [25]. In Burkina Faso, the spatial distribution of the snail host is determined by the strong South-to-North climatic gradient of the transition from sudanian to sahelian regimes [26]. An extensive study of the ecology of both *Bulinus spp*. and *Biomphalaria pfeifferi* in Burkina Faso revealed both the importance of climatic zones and habitat type (including natural and man-made) on snail presence or absence [27]. Indeed *B. pfeifferi* was seldom found North of the 12th parallel and absent North of the 14th [28], matching the observed historical range of intestinal schistosomiasis in the country [21]. Furthermore, a marked climatic seasonality in Burkina Faso determines the ephemerality of waterbodies and hydrological networks, hence of potential transmission, thus strongly conditioning several epidemiological characteristics of the disease [20]. Not only do climatic and environmental conditions greatly limit the ecological ranges of the intermediate hosts of the disease, but they also underpin the patterns of human-water contacts during socio-economic and domestic activities through which both exposure to infection and contamination of waterbodies occur [29–32]. In Burkina Faso agricultural activities (market gardening or *maraîchage*, rice culture) and fishing (in reservoirs or rivers) are the main causes of male human-water contacts, whereas domestic activities (laundry, dish washing, water collection) are the main factors of exposure to infection for women [32, 33]. Contamination and exposure for children are known to be mainly associated with recreational bathing in streams, temporary ponds and reservoirs, and participation in parental agricultural activities [29, 33]. Seasonal variation of human-water contacts has not yet been addressed Burkina Faso to our knowledge. Even rudimentary notions of disease transmission mechanisms and prevention have been reported to be poor in most of the settings that have been investigated [21, 29, 32].

Human mobility is known to play a major role in the persistence and the expansion of the disease within and from endemic areas in Africa [34–37] and Brazil [38]. Indeed people may become infected while performing their livelihood tasks away from home and importing the disease back in their home village. Furthermore infected migrants coming form endemic regions can introduce schistosomiasis in disease-free villages. This type of medium-to-long range contamination is indeed compatible with the successive focal transmission of the disease at the local level suggested by recent landscape genetics studies [39]. The socio-ecological and epidemiological specificities of the transmission of intestinal schistosomiasis in sub-Saharan Africa and Brazil may explain the different roles played by human mobility, a topic to be elucidated by future research. Given its importance for transmission in the African context, data regarding mobility patterns, possibly directly via cellular phone displacements, will be necessary to produce reliable predictive tools for disease elimination both in terms of re-appearance and of prioritization of directed interventions in Burkina Faso.

**Fig 1:**
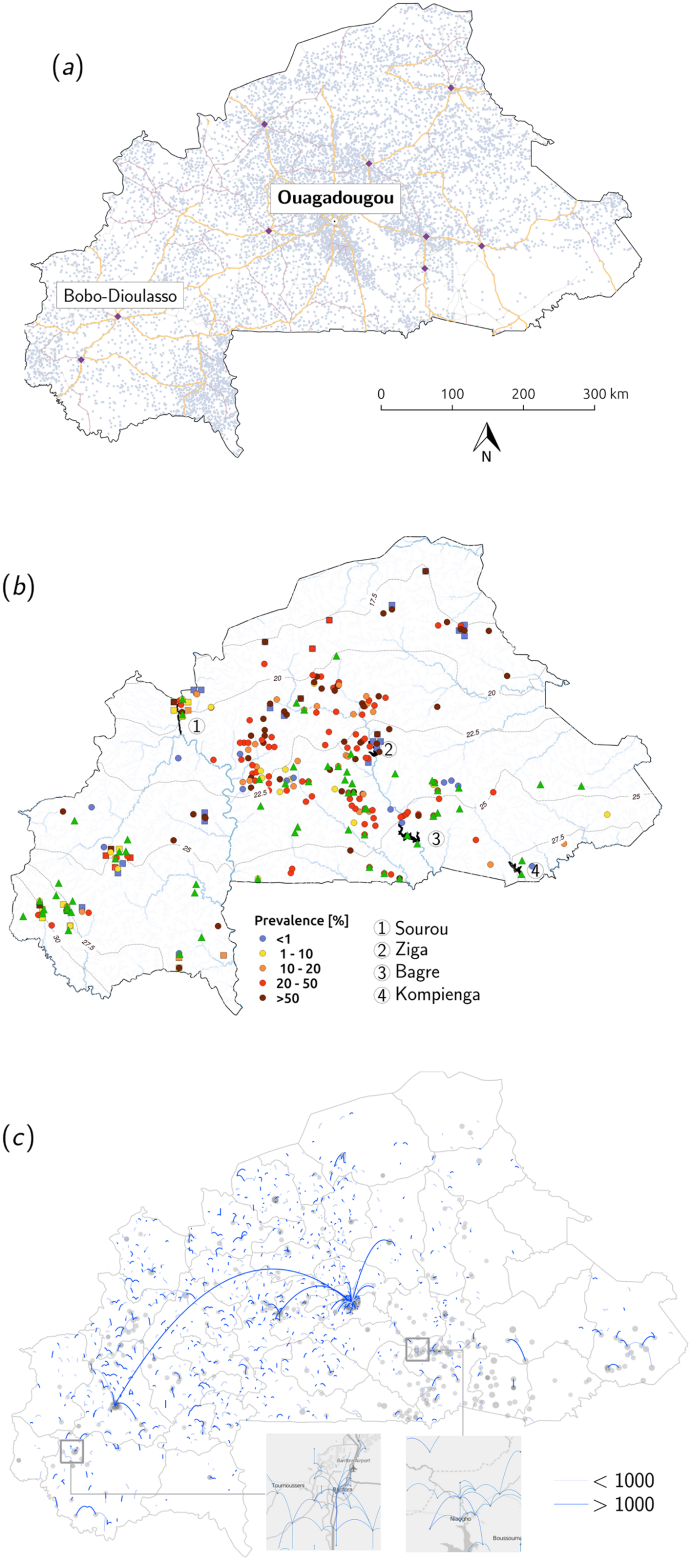
Model implementation for Burkina Faso. (a) Road network and settlement distribution. The principal transportation axes, the main cities (black points), including the capital, Ouagadougou and, the regional hub, Bobo-Dioulasso, and smaller settlements (grey) are represented. (b) Water resources and schistosomiasis. Only the four largest dams of the country are shown (numbered 1–4) along with the major rivers and mean monthly precipitation isolines (isohyets). Historical prevalence of intestinal(urinary) schistosomiasis is represented in color-coded rectangles(circles). Data consists of published parisotological studies in Burkina Faso from 1955 to 2007 (the average prevalence over survey years was taken in villages where multiple surveys were available). Green triangles represent *B. pfeifferi* presence data compiled by [27]. (c) Human mobility patterns Fluxes were predicted using the radiation model and the population density of each node. Point size is proportional to the log_10_ of settlement population. Fluxes are divided into higher and lower than 1′000 travellers (evaluated taking all of people in the node travelling) and displayed in the main figure for settlements larger than 5′000 people. The tow insets correspond to zooms on areas in the South-West and the East of the country with all fluxes larger than 1′000 travellers depicted. Spatial data sources are given in S1 Table. Inset maps produced with [77].

As observed in many other schistosomiasis-affected countries, agricultural development and the construction of large-scale irrigation schemes have induced anthropogenic perturbations of the underlying natural matrix affecting schistosomiasis distribution [37, 40–44]. This fact is well illustrated in Burkina Faso by the construction of the Sourou valley dam at the northern border with Mali in the late 1980’s (Fig 1), which resulted in the expansion of the ecological range of *Biomphalaria pfeifferi* along the Mouhoun river from the Bobo-Dioulasso region to the north [21, 42]. Arrival of intestinal bilharzia followed shortly, possibly brought in by migrants coming from the endemic South of the country to work in the rice paddies, yielding an increase in prevalence from virtually zero to more than 60% among school-aged children in villages located around the Sourou dam in about 10 years. With a 5-fold increase in the number of small reservoirs in Burkina Faso in the past 60 years, and in the perspective of further constructions of large dams [45], these observations highlight the need to explicitly address the impact of water resources development on schistosomiasis transmission [43, 46]. The interwoven complexity of economic development, water management, and ecology make the need for appropriate prediction tools to attain an integrated control of schistosomiasis [47] addressesing the inherent contradiction between water resources development and livelihood preservation [48, 49] linked to the emergence and persistence of waterborne diseases [44]. Under this viewpoint large improvements can be made in terms of both model adequacy to local contexts, in this case that of the country of Burkina Faso, and knowledge of the processes related to disease transmission, specifically human mobility and snail ecology.

Mathematical models have proved useful in analysing the spread of both micro- and macro- parasitic waterborne diseases in spatially explicit settings e.g. [11–15]. Furthermore social and hydrological connectivity for schistosomiasis have been discussed in the context of distributed human-snail contact sites [50, 51], and formally developed in agent-based [52] and connected metapopulation models, building on the seminal Macdonald schistosomiasis model [23]. Gurarie and Seto specifically highlighted the potential importance of both hydrological and social connectivity in small-scale environments [12]. Similar in their approach, spatially explicit metapopulation models have been developed for waterborne diseases such as cholera for which a generalized framework has been developed for determining spatially explicit invasion conditions and predicting spatial patterns of disease spread [53–55]. The exploration of invasion conditions could be of relevance in the perspective of sustainable elimination of schistosomiasis, and the inference of the spatial scale of the deployment of control measures in changing conditions of endemicity. The transition between morbidity-centred control programs and transmission-based elimination strategies would ideally require the deployment of surveillance-response mechanism entailing a higher degree of planning and decision-making than traditional mass drug administration campaigns [56], thus calling for new tools to support them [57]. The design of improved sampling protocols for surveillance, and the investigation of transmission breakpoints have been recently highlighted as research priorities for helminth diseases modelling [57]. In line with such priorities, we propose a novel mathematical exploration of an established model [12] and an application of the geography of the disease to the context of Burkina Faso. Special emphasis is placed on linking disease spread and human mobility and water resource development as a proof of concept for the usefulness of the implementation of these kind of tools for macroparasitic waterborne diseases like schistosomiasis.

## Materials and Methods

### The model

The mechanistic process model proposed by [12] expanded the seminal spatially implicit model of schistosomiasis by [23]. The extension proposes a connected metapopulation network of *n* villages. The system of differential equations is expressed in terms of the mean worm burden in human populations, *W*_*i*_, the prevalence of infection in the snail intermediate hosts, *Y*_*i*_, and the densities of cercariae and miracidia, *C*_*i*_ and *M*_*i*_, the two intermediate larval stages of the parasite. Connectivity is accounted for by human mobility and hydrologic transport of larvae (while neglecting snail mobility). The parameters of this model are explained in Table 1. With our notation, the model of [12] can be written as:

$$\begin{array}{cc} \frac{dW_{i}}{dt} & =a\;[\left(1-m_{i}\right)\theta_{i}C_{i}+m_{i}\sum_{j=1}^{n}Q_{ij}\theta_{j}C_{j}]-\gamma W_{i}\\ \frac{dY_{i}}{dt} & =bM_{i}\left(1-Y_{i}\right)-\nu Y_{i}\\ \frac{dC_{i}}{dt} & =\frac{\Pi_{C}}{V_{i}}N_{i}Y_{i}-\mu_{C}C_{i}-l_{i}^{C}C_{i}+\sum_{j=1}^{n}l_{j}^{C}P_{ji}S_{ji}^{C}\frac{V_{j}}{V_{i}}C_{j}\\ \frac{dM_{i}}{dt} & =\frac{\Pi_{M}}{V_{i}}\theta_{i}^{\prime }\;[\left(1-m_{i}\right)H_{i}\frac{W_{i}}{2}+\sum_{j=1}^{n}m_{j}H_{j}\frac{W_{j}}{2}Q_{ji}]-\mu_{M}M_{i}\\ & \;\;\;-l_{i}^{M}M_{i}+\sum_{j=1}^{n}l_{j}^{M}P_{ji}S_{ji}^{M}\frac{V_{j}}{V_{i}}M_{j}\; \end{array}$$ (1)

**Table 1:** Model parameter description and values used in analysis of intestinal schistosomiasis in Burkina Faso.

		description	value	ref.
state variables	*W*_*i*_	mean worm burden in village *i*	-	-
	*Y*_*i*_	infection prevalence in snail population in node *i*	-	-
	*C*_*i*_	cercarial density (per unit volume) in node *i*	-	-
	*M*_*i*_	miracidial density (per unit volume) in node *i*	-	-
demographic and ecological parameters	*H*_*i*_	human population in node *i*	census	[68]
	*N*_*i*_	snail population in node *i*	inferred	[69]
	*V*_*i*_	water volume in node *i*	1	-
	*a*	probability of successful cercarial infection in humans	10^−5^	[70]
	*b*	probability of successful miracidial infection in snails	5 × 10^−5^	[70]
	*θ*_*i*_, $\theta_{i}^{\prime }$	local exposure and contamination rates	range	[12]
	Π_*C*_	cercarial emission rate/infected snail	100 [day^−1^]	[70]
	Π_*M*_	miracidial emission rate per worm pair	300 [day^−1^]	[70]
	*μ*_*C*_	per capita mortality rate of cercariae	0.91 [day^−1^]	[24]
	*μ*_*M*_	per capita mortality rate of miracidia	3.04 [day^−1^]	[24]
	*γ*	per capita mortality rate of schistosome in host	1/5 [yr^−1^]	[24]
	*ν*	per capita mortality rate of snails	1/0.1 [yr^−1^]	[24]
connectivity parameters	*m*_*i*_	fraction of moving residents of node *i*	range	-
	*Q*_*i*,*j*_	human mobility probability matrix of a traveler moving from *i* to *j* and back	inferred	[71]
	$l_{i}^{C},l_{i}^{M}$	hydrologic throughput rates of cercaria and miracidia	not used	-
	*P*_*ji*_	hydrologic connectivity matrix (0/1 entries)	not used	-
	$S_{ji}^{C},S_{ji}^{M}$	= *e* ^−*β_C,M_d_ij_*^; transport survival rates of cercariae and miracidia between nodes separated by distance *d*_*ij*_.	not used	-
	*β*_*C*,*M*_	larval hydrologic survival exponents during transport	not used	-

Here at each node *i*, the rate of change in the mean worm burden is given by the difference between the intensity of cercarial infection expressed as a probability of infection upon contact *a* times the exposure to contaminated water *θ*_*i*_ and cercarial density, and worm mortality at given rate *γ*. Density of cercariae is dynamically determined by the *per-capita* cercarial output from infected snails Π_*C*_ and the number of infected snails *N*_*i*_ *Y*_*i*_ at time *t*, and limited by cercarial mortality *μ*_*C*_. The rate of change in the prevalence of infected snails is given by the probability of infection of a susceptible snail *b*(1 − *Y*_*i*_) times miracidial density and limited by snail mortality *ν*. Finally, miracidial density is determined by the rate of miracidial output from humans Π_*M*_, the contamination rate $\theta_{i}^{\prime }$ and the number of pairs of adult schistosomes, given by half of the mean worm burden times the number of human hosts *H*_*i*_, divided by the volume of water *V*_*i*_ in which they are released (to obtain larval concentration in water). Human mobility is included by specific contact distribution fractions *Q*_*ij*_. In the original version of [12], the connectivity matrix is set to be proportional to distance between nodes, i.e. *Q_ij_* ∝ *e* ^−*αd_ij_*^ with *α* being a measure of zonal social connectivity. Hydrologic connectivity is implemented through a distance-dependent transport survival rate *S_ij_* = *e* ^−*βd_ij_*^ for each larval stage from upstream to downstream settlements with different volumes of contaminated water *V*_*i*_. Model 1 differs slightly from the one proposed by [12] in the way it treats larval densities and human connectivity. Indeed, we explicitly introduce the volume of water *V*_*i*_ in which larvae are released in order have the state variable expressed as a concentration per unit volume (rather than general unit habitat as expressed by [12]). However, in our analysis we set all *V*_*i*_ to 1 so as restrain the analysis on the connectivity parameters. Regarding human connectivity, we choose to keep traveller contacts and local contacts distinct by the mobility parameter *m* instead of distributing the whole local contact parameters *θ*_*i*_ as a function of population and distance as proposed by [12] in their formulation of a social contact matrix. Indeed in their approach *m*_*i*_ is implicitly considered to be constant and proportional to *H_i_*/ ∑_*j*_ *H_j_e* ^−*αd_ij_*^ for local contamination and exposure rates. By distinguishing between travellers and non-travellers we assume that contamination and exposure rates depend only on the physical characteristics of the site, and not on the traveller origin (captured by *θ*_*i*_). This is reasonable when analysing patterns on a national scale where the physical characteristics of human settlements are heterogeneous (cites vs. towns vs. villages), and thus the contact rate at a visited location *θ*_*j*_ depends mainly on *j*. The main features of Model 1 are (1) well-mixed and stationary human and snail populations at each village *i* (2) miracidial dispersal and cercarial uptake through human movement matrix *Q*_*ij*_ modulated by the fraction of moving people *m*_*i*_, and (3) miracidial and cercarial dispersal through hydrologic connectivity matrices $P_{ji}S_{ji}^{C}$ and $P_{ji}S_{ji}^{M}$ modulated by the hydrologic transport rates $l_{j}^{C,M}$. By setting $\{m_{i},l_{j}^{C,M}\}=0\;\forall i$, *j* Model 1 collapses to the local model of [23] expressed for each individual settlement, for which the local basic reproduction numbers are derived as $R_{0,i}=\left(ab\theta \theta^{\prime }\Pi_{C}\Pi_{M}H_{i}N_{i}\right)/\left(2\gamma \nu \mu_{C}\mu_{M}V_{i}^{2}\right)$ (see S1 Text). In this simple case, parasite invasion of the disease-free system depends locally on the condition *R*_0,*i*_ > 1. The above-mentioned model [12] does not include detailed biological controls (worm mating probability, negative density feedback on within-host worm population) nor immunological ones (acquired immunity of human hosts). Although this could prove inadequate for the study of a particular village close to endemic equilibrium, we deem these factors of secondary importance in the analysis of pathogen invasion conditions at the national level. The stability analysis of the model is carried out here for our reformulated version and extended to explore the spatial patterns of disease spread. Sensitivity analysis of model assumptions can be found in [12].

### Parasite invasion conditions

Despite the complexity given by the spatially explicit formulation of Model 1, a rigorous stability analysis can be used to determine spatially explicit conditions for pathogen invasion. As the system is positive and the disease-free equilibrium is characterized by null values of the state variables, the bifurcation can only occur via an exchange of stability. Specifically, the disease-free equilibrium switches from stable node to saddle through a transcritical bifurcation, at which the Jacobian has one zero eigenvalue [12, 53, 58, 59]. Parasite invasion is determined by the unstability of the disease-free equilibrium (DFE) **X**_**0**_ = [**0**_*n*_, **0**_*n*_, **0**_*n*_, **0**_*n*_]^*T*^, with **0**_*n*_ a 1 × *n* null vector, *n* being the number of settlements. The stability properties of the DFE can be studied by analysing the Jacobian linearised at the DFE, $J_{0}^{*}$, which reads:

$$J_{0}^{*}=[\begin{array}{cc} A & B\\ C & D \end{array}]\;,$$ (2)

where

$$A=[\begin{array}{cc} -\gamma I & 0\\ 0 & -\nu I \end{array}]\;B=[\begin{array}{cc} a(I-m+mQ)\theta & 0\\ 0 & bI \end{array}]$$

$$C=[\begin{array}{cc} 0 & \Pi_{C}V^{-1}N\\ \frac{\Pi_{M}}{2}V^{-1}\theta^{\prime }\left(I-m+Q^{T}m\right)H & 0 \end{array}]$$

$$D=[\begin{array}{cc} -\mu_{C}I+T_{C} & 0\\ 0 & -\mu_{M}I+T_{M} \end{array}]\;.$$

where: **I** is the identity matrix; **m**, ***θ***, **V**, **N**, ***θ*′** and **H** are diagonal matrices whose non-zero elements are made up by the parameters *m*_*i*_, *θ*_*i*_, *V*_*i*_, *N*_*i*_, $\theta_{i}^{\prime }$ and *H*_*i*_, respectively; **Q** = [*Q*_*ij*_] is the connectivity matrix for human mobility; **T_C_** = (**V** ^−1^ **P**_**C**_^*T*^ **V** − **I**) **l_C_** and **T_M_** = (**V** ^−1^ **P**_**M**_^*T*^ **V** − **I**) **l_M_**, while $P_{C}=\left[P_{ij}S_{ij}^{C}\right]=P\circ S_{C}$ (where ∘ is the Hadamard product) and $P_{M}=\left[P_{ij}S_{ij}^{M}\right]=P\circ S_{M}$ are the transport matrices accounting for hydrologic connectivity and larval survival during transport, and $l_{C}$ and $l_{M}$ are diagonal matrices whose non-zero elements are the local values of $l_{i}^{C}$ and $l_{i}^{M}$, respectively. After some manipulations (see S1 Text), the bifurcation condition $\det(J_{0}^{*})=0$ can be reformulated as:

$$\det(I-{\left(I-m\right)}^{2}R_{0}+R_{0}^{M}\left(m,Q\right)+T\left(\mu_{C},T_{C},\mu_{M},T_{M}\right))=0$$ (3)

We can define a generalized reproduction matrix (GRM) for our model of schistosomiasis transmission as the sum of three matrices. One depends on local dynamics only; the other two (non-linearly) on spatial coupling mechanisms, reading,

$$G_{0}={\left(I-m\right)}^{2}R_{0}+R_{0}^{M}\left(m,Q\right)+T\left(\mu_{C},T_{C},\mu_{M},T_{M}\right)\;.$$ (4)

Parasite invasion conditions can therefore be decomposed into spatially explicit local conditions, **R_0_**, and connectivity-induced conditions $R_{0}^{M}$ and **T** for mobility and hydrologic transport respectively. **R_0_** is a diagonal matrix, whose non-zero elements are the local values *R*_0__*i*_ of the basic reproduction number. $R_{0}^{M}$ is a matrix depending on human mobility structure and magnitude, i.e.

$$R_{0}^{M}\left(m,Q\right)=\frac{ab\Pi_{C}\Pi_{M}}{2\gamma \nu \mu_{C}\mu_{M}}NV^{-1}\theta^{\prime }[\left(I-m\right)mHQ+Q^{T}Hm\left(I-m\right)+Q^{T}m^{2}HQ]\theta V^{-1}.$$

**T** is another matrix depending on larval death rates and transport through the hydrologic network

$$T\left(\mu_{C},T_{C},\mu_{M},T_{M}\right)=\frac{1}{\mu_{C}}VT_{C}V^{-1}+\frac{1}{\mu_{M}}NT_{M}N^{-1}-\frac{1}{\mu_{C}\mu_{M}}NT_{M}VN^{-1}T_{C}V^{-1}.$$

Ensuing from Eqs 3 and 4 the condition for parasite invasion can be stated in terms of the dominant eigenvalue *g*_0_ of matrix **G_0_**, i.e. *g*_0_ = max_*k*_(*λ*_*k*_(**G_0_**)), with disease spread occurring if *g*_0_ > 1. Bearing the operational use of this condition in mind, it is important to note that control measures that successfully lead to a stable DFE (driving *g*_0_ below 1, for instance by improving sanitation or through snail control [60]) do not imply immediate transmission interruption, i.e. non-zero parasite loads in the population and in the environment persist for some time. In ecological terms, the time to reach pathogen extinction corresponds to the relaxation time of the system [61–63], $t_{R}$, which is determined by the characteristic timescale of system trajectories approaching the DFE. The relaxation time of schistosomiasis transmission is therefore set by the dominant eigenvalue *g*_0_ as $t_{R}=1/\left|\log\left(g_{0}\right)\right|$. If *g*_0_ < 1 parasite transmission decreases exponentially in time. Therefore, transient dynamics can safely be assumed to be over after $5t_{R}$ time units. Based on this relation it is possible to associate a given socio-ecological setting in the country (i.e. a given value of *g*_0_) to an estimate of the time for transmission to effectively die out once conditions for pathogen extinction are met (*g*_0_ < 1).

Eigenvector analysis, here carried out for the first time on spatially explicit modes of schistosomiasis, proves capable of capturing key features of the spatial pattern of empirically reported infections/prevalences (see e.g. [54]). The dominant eigenvector **g_0_** of the GRM can be used to study the geography of disease spread close to the transcritical bifurcation. Here, **g_0_** corresponds to the cercarial component **C** of the vector of state variables for trajectories diverging from the (unstable) DFE (see S1 Text). This is of particular interest in the study of spatial patterns of disease spread. In fact, close to an unstable DFE the dominant eigenvector of matrix J_0_*, $\lambda_{\text{max}}\left(J_{0}^{*}\right)$, pinpoints the directions in the state space along which the system trajectories will diverge from the equilibrium. In other words, computing $\lambda_{\text{max}}\left(J_{0}^{*}\right)$ enables not only the quantitative analysis of conditions for the parasite to invade a connected set of spatial locations, but also to predict the direction of disease spread geographically in the time close to the onset. Of particular interest in the dominant eigenvector analysis is the mean worm burden compartment, **W**, which in the case of schistosomiasis is an important measure of infection severity and the natural target of control interventions through antischistosomal treatments [64, 65]. At the bifurcation through which the DFE loses stability, spatial distribution of the mean worm burden can be computed in terms of **g_0_** as **W** = (*a*/*γ*) (**I** − **m** + **mQ**)***θ*g_0_** (see S1 Text).

### Model implementation

Schistosomiasis control and elimination programs are implemented on the national scales by centralized national institutions [56]. We apply the dominant eigenvalue and eigenvector analysis to the context of Burkina Faso, a landlocked country in the transition zone from the sudanian to the sahelian climatic zones [26], emblematic of the intertwined ecological, climatic and soci-economic drivers of schistosomiasis transmission and persistence [21]. Schistosomiasis has been known in the country since the first demographic surveys in the 1950’s [21] and has yet to be eliminated despite national-scale control measures from 2004–2008 by the Schistosomiasis Control Initiative [19, 66, 67]. Both urinary and intestinal forms of the disease are present with very different historical geographical coverages, governed by the ecological ranges of their respective intermediate hosts [21]. The intestinal form of the disease will be used for the subsequent analysis given the proven importance of human mobility in its geographical expansion in the country [20].

The parameters of Model 1 and the values used for its implementation are detailed in Table 1. Sources and references of the spatial data used in the analysis are given in S1 Table.

#### Settlements and population—*H*_*i*_

A settlement is a geographic location having any number of permanent residents. The population of the settlements in Burkina Faso ranges from villages of less than 100 inhabitants to cities of more than 100’000 people, the urban area of the capital Ouagadougou housing more than 700’000. A settlement was considered to be a city if its population *H*_*i*_ is bigger than a definition threshold of 10’000 inhabitants, and a village if *H*_*i*_ is smaller than 2’000 inhabitants. The model was applied to 10′592 settlements. Population was obtained by assigning the sum of gridded Landscan estimates of population density (S1 Fig) to the Voronoi polygons resulting from the spatial configuration of settlements.

#### Intermediate host distribution—*N*_*i*_

The intermediate host of intestinal schistosomiasis in Burkina Faso is an aquatic gastropode named *Biomphalaria pfeifferi*. The ecological range of the species is determined by both climatic and hydrologic conditions, namely the presence of agricultural infrastructure such as small and large reservoirs [42–44]. Presence and abundance of the host determine the viability of the transmission cycle of the disease. In the mathematical framework proposed by [12], the number of snails in a given node, *N*_*i*_, is given. Detailed snail counts at the settlement level in the country of Burkina Faso are ongoing in a number of villages, and are beyond the scopes of this study. For surveillance-response programs at the national level, a probability of presence is retained as a proxy of snail abundance. Probability of presence can be modelled for entire landscapes based on underlying environmental covariates, preserving information about heterogeneity across sites. The Maximum Entropy (MaxEnt) approach by [69] is one of such methods that has proven its worth in comparison to other predictive methods, and has already been used in the specific case of modelling the distribution of the intermediate hosts of the two genus of schistosomes across Africa [72]. The MaxEnt framework is based on the distribution of presence records versus continuous environmental covariates, specifically species distribution is modelled by maximizing its distribution entropy, subject to the constraint of having the same expected value of each environmental variable than its empirical average [73].

Available data for *B. pfeifferi* to implement MaxEnt modelling consisted of presence data obtained from the comprehensive literature review and field work of [27] in Burkina Faso, including a total of 64 unique sighting locations. For a detailed description of the sampling methodology we refer to [27]. In brief, standardized local samplings were performed throughout the country between 1984 and 1993 and routine identification protocols were used for species discrimination. The number of sites characterized is indeed remarkable (for a comparison with other African sites see e.g. [72]). Modelling was performed in the freely available software provided by [69]. Selection of features and natural environmental covariates for prediction was done according to the method proposed by [72]. The only anthropogenic covariate used was the distance to dams or small reservoirs, $D_{water}$, which was produced by calculating the euclidean distance from each settlement to the closest water surface. Water surfaces were obtained at a 30m pixel resolution for all of Burkina Faso by Quadratic Discriminant Analysis of a mosaic of Landsat satellite images of 2014 (courtesy of the U.S. Geological Service). The resulting water surfaces were filtered and validated against the national spatial database of dams and small reservoirs [46]. Maps of the covariates used as inputs for MaxEnt are illustrated in S2 Fig, and their relative importance for modelling the ecological range are presented in S3 Fig. Interestingly the variable with both the most unique information and the most explanatory power was found to be the distance to reservoirs, thus supporting the need for investigating the role of water resources development in schistosomiasis transmission. The site-specific parameter *N*_*i*_ was taken as being proportional during model implementation to the output of MaxEnt (probability of presence).

#### Human mobility model—*Q*_*ij*_

No actual human mobility data, such as census or mobile phone records, are available for accurately estimating human mobility fluxes in Burkina Faso. Models of human mobility have grown popular and accurate with increasing access to big data on human behaviour, and are valuable tools to overcome data scarcity despite the strong assumptions that need to be made regarding, for instance, travel means and accessibility [74, 75]. Here we implement the recently proposed radiation model which has proven to correctly reproduce mobility patterns at the national and regional scales originally implemented in the context of the USA [71], and proved to hold well for inter-city movement in West Africa [76]. Specifically, the radiation model expresses the probability *Q*_*ij*_ that a person travelling out of node *i* reaches node *j* as *Q*_*ij*_ = *H*_*i*_ *H*_*j*_/{(*H*_*i*_ + *s*_*ij*_) (*H*_*i*_ + *H*_*j*_ + *s*_*ij*_)}, where *H*_*i*_[*H*_*j*_] is the population size of the origin [destination] node, and *s*_*ij*_ is the total population living within a radius *d*_*ij*_ around the origin, excluding the origin and destination populations, and *d*_*ij*_ being the distance between nodes *i* and *j*. The model has therefore the benefit of depending explicitly only on population density distribution in space to capture the structure of human mobility fluxes. The fraction of moving people, i.e. people visiting another node during a period of time, *m*_*i*_, is therefore the only free parameter modulating the strength of mobility-related connectivity in the spatial spread of the disease. To our knowledge, no empirical estimate of *m*_*i*_ exists for Burkina Faso, although values of *m*_*i*_ → 1 are unlikely. Due to the lack of information on the heterogeneity of mobility habits in Burkina Faso we assume that the fraction of moving people is not site-dependent, thus dropping the subscript *i*. The vector of human population **H** and Euclidean distances were used to produce a 10′592 × 10′592 matrix *Q*_*ij*_ of human mobility connections. Three forms of human mobility can be observed in Fig 1: (1) strong local-level fluxes between low density populated areas, (2) large population centres interconnected by long-distance trips, and (3) medium-range fluxes in the basin of attraction of the two major cities (Ouagadougou and Bobo-Dioulasso).

#### Exposure and contamination rates—*θ_i_*, $\theta_{i}^{\prime }$

Successful human infection by cercariae strongly depends on water contact frequency and duration, which in turn are determined by the socio-economic activities of the populations [32, 37]. For this reason, urban areas are potentially less favourable schistosomiasis transmission sites given the lower human-to-water contact opportunities relative to the number of inhabitants, which needs to be encompassed into the single $\theta_{i}\left[\theta_{i}^{\prime }\right]$ parameters of exposure[contamination] rate for the whole population of a node *i*. Although there have been examples of recent increases in agriculture around small reservoirs and other water bodies in urban areas [78, 79], the proportion of such contact patterns is not deemed representative of the majority of any urban settlement [37, 80]. In the absence of detailed and nation-wide socio-economic surveys assessing exposure and contamination rates in Burkina Faso, we have translated the reduced probability of having population-wide high contamination and exposure rates in urbanized settlements into an inverse-logistic formulation for the exposure/contamination rates (here assumed to coincide, i.e. *θ* = *θ*′, as in [12]) based on the size of each node. The underlying hypothesis is that in Burkina Faso densely populated urban centres have low contamination/exposure rates with respect to small rural settlements where a large fraction of socio-economic activities entail human-contaminated water contacts [31, 32]. The resulting contact rate *θ*_*i*_ in node *i* is thus,

$$\theta_{i}=\theta_{MAX}\;\{1-\frac{1}{1+u_{\alpha }e^{-v_{\alpha }Z_{i}}}\},$$ (5)

that is a sigmoid decreasing function of population size. Here *θ*_*MAX*_ is a parameter indicating the maximum contact rate, while *u*_*α*_ and *v*_*α*_ are two positive coefficients that control the shape of the *θ*_*i*_ function. Specifically the coefficients *u*_*α*_ and *v*_*α*_ are computed so that cities, here taken to be defined as settlements of 10′000 inhabitants, have exposure equal to *αθ*_*MAX*_, and imposing *θ*_*i*_ = 0.9 ⋅ *θ*_*MAX*_ for villages of exactly 2′000 inhabitants. The function form *θ*_*i*_ therefore only depends on population in location *i* and one free parameter, *α*, that can be seen as the percentage reduction in exposure/contamination rates due to urbanization for a settlement at the population threshold defining a city with respect to the maximal rates observed in rural areas. *Z*_*i*_ = (log_10_(*H*_*i*_) − *μ*_log_10_(**H**)_)/(*σ*_log_10_(**H**)_) is the log_10_-transformed and standardized population density based on the mean, *μ*_log_10_(**H**)_, and the standard deviation, *σ*_log_10_(**H**)_, of the log10 transform of the population vector **H**. Illustrations of the resulting functional forms for different values of *α* are given in S4 Fig. Note that the above parametrization, meant to tackle the upscaled model of exposure, are by no means claimed to be the best possible choice. The choice of Eq 5 was made with the objective of discriminating between the very different settings of urban areas and rural settlements, and that other functional forms could have been chosen that respect the low vs. high contamination/exposure rates. Indeed the inclusion of small-scale heterogeneity between settlements of similar sizes (for which Eq 5 would predict similar contact/exposure rates) depends on the presence and density of improved sources of water and sanitation infrastructure, as well as the ephemerality of the hydrological network and waterbodies, and on other factors for which detailed geo-referenced data are not readily accessible. Furthermore this approach clearly lacks the accuracy of water contact surveys or direct observations of human-water contacts such as the ones provided for particular sites across Sub-Saharan Africa [40, 81], but at least enables a relative ranking of nodes at the national scale following the hypothesis that urbanization leads to a reduction in human-water contacts. A more accurate generalizable modelling of human-water contacts based on water and sanitation infrastructure data and resulting contamination and exposure rates for schistosomiasis would be of great use in reducing the uncertainty related to this particular point.

#### Hydrologic connectivity

In Eq 1, hydrologic connectivity only concerns larval stages of the parasite. Survival times of both miracidia and cercariae is known to be on the order of hours [82], thus resulting in localized effects of this form of transport, as illustrated in [12] for a their network of 15 connected villages. Since the context of the present analysis is all of Burkina Faso, we deemed it realistic to remove hydrologic transport of the larval stages of the parasite, since the only viable hydrologic transport routes at the national scale could be large rivers (Fig 1), which are known to be unsuitable environments for larvae that have limited life spans. Nevertheless, all of the following analyses could be repeated including hydrologic transport by applying Eq 4 in its full version. On the other hand, a form of hydrologic connectivity that was not considered in [12] is the passive dispersion of the snail intermediate host in streams and rivers. Indeed it has been observed that water velocities greater than approximately 33cm/s may dislodge *Bulinus spp*. individuals that may be transported downstream [27]. The only available data of this kind in the fairly flat topography of Burkina Faso is a study by Poda [27] which measured snail dispersion during the rainy season on the Nazinon river (Volta Rouge). No information was collected on snail provenance, transport distance and variability between and among species. We considered that there was not enough evidence to justify modifying Eq 1 to include snail transport at this stage. Field data on the transport and ecology of the intermediate hosts of schistosomiasis in Burkina Faso is ongoing to elucidate its relevance in large-scale transmission models for the context at hand.

## Results and Discussion

### Pathogen invasion conditions

**Fig 2:**
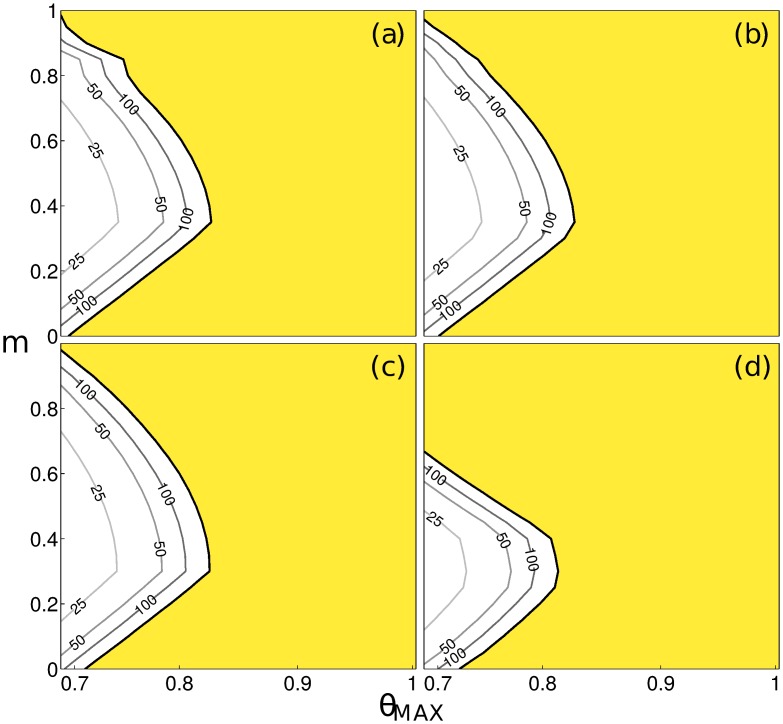
Pathogen invasion conditions and average time to extinction for the Burkina Faso settlement network. The values of the dominant eigenvalue *g*_0_ of the GRM are plotted for increasing values of *α* ∈ {0.0001, 0.001, 0.01, 0.1} (respectively in panels (a) to (d)), against values of maximal contamination and exposure rates *θ*_*MAX*_ and the fraction of mobile people *m*. Yellow indicates parameter combinations permitting parasitic invasion. Contours to the left of the stability boundary give pathogen extinction time isolines ($5t_{R}$) of 25, 50 and 100 years.

Because the real magnitude of human mobility in Burkina Faso is currently unknown, we explore a wide range of possible scenarios. We assume that the structure of the human mobility network is accurately captured by the radiation model, thus we explore only the intensity of mobility fluxes. In order to encompass all possible behaviours in real large scale mobility patterns (although extremes are unlikely) values of the fraction of mobile population in the range *m* ∈ [0, 1] are considered. The relation between contamination and exposure rates and population density dependence also lacks ground-truthed data, and therefore it is explored through sigmoid contact functions (Eq 5) with *α* ∈ {0.0001, 0.001, 0.01, 0.1}. Increasing *α* can be seen as increasing urban contamination and exposure rates. Not surprisingly, human mobility is found to strongly condition successful parasitic invasion of the system. Two opposite effects are illustrated in Fig 2 with a dilution effect for fractions of mobile people increasing from *m* = 0 to *m* ≈ 0.3; and an increase in the risk of disease spread for *m* > 0.3. Indeed for all investigated levels of urban contamination and exposure rates the dominant eigenvalue *g*_0_ of the GRM falls below 1 for intermediate ranges of the fraction of mobile people and low values of *θ*_*MAX*_. Beyond the specificities of Model 1, these results are consistent with the conclusions of [12], at least close to the bifurcation and for the parameter region where *m* < 0.5. The parameter region in which the DFE is stable (gray shaded area in Fig 2), thus preventing parasite invasion, is of similar sizes for all values of *α*, except for high levels of urban contact rates (*α* = 0.1) for which pathogen invasion is observed for a wider range in the parameter space. This can be seen as the effect of coupling locations with high mobility (densely populated areas) with locations with high transmission potential (high contamination and exposure rates). For all subsequent analysis we therefore focused on the case that seemed the most realistic, namely intermediate urban contamination and exposure rates (*α* = 0.01).

In addition to the stability of the DFE, the dominant eigenvalue of the GRM contains information on the average time for transmission to fade out in the case of unfavourable conditions for pathogen spread. The relaxation time isolines (constant $t_{R}$) follow the shape of the stability boundary, with a logarithmic decrease of extinction time as a function of distance to the boundary *g*_0_ = 1. The time to extinction is of the order of a century close to the stability line, and decreases sharply for small modifications of human mobility and contamination/exposure rates. Similarly to the stability conditions, the relaxation time is minimal at intermediate mobility levels for a given *θ*_*MAX*_. Furthermore the shape of the time to extinction isolines suggests that above the mobility threshold, say *m* ≈ 0.3, reducing contamination/exposure rates would have a larger marginal reduction on the time to extinction than acting on mobility.

### Predicted spatial patterns

#### Proof of concept for macro-parisitic dynamical systems

**Fig 3:**
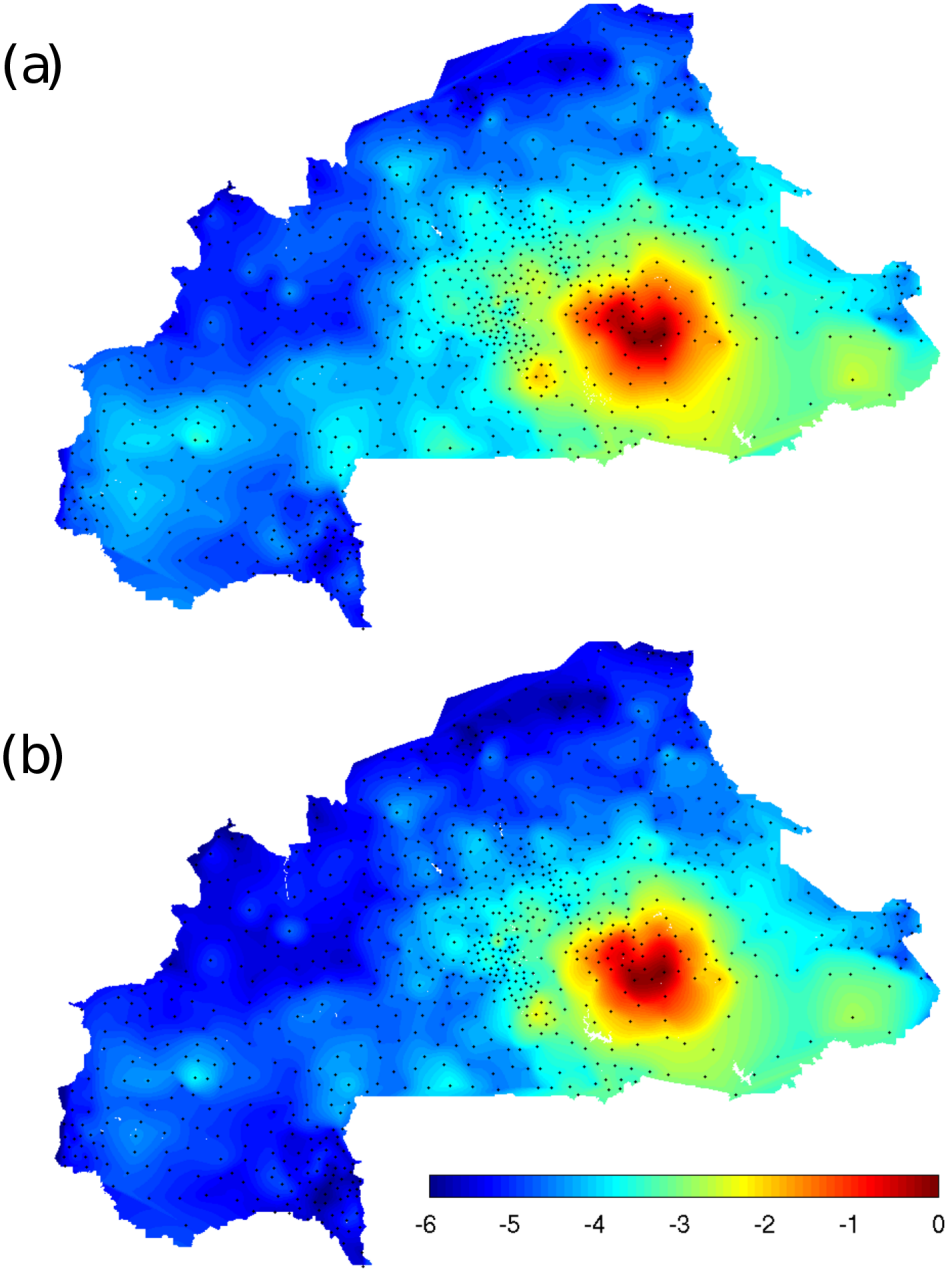
Eigenvector analysis as predictive tool. Comparison of the equilibrium state of the system for a 1′000 node aggregated network for the parameter set {*α* = 0.01, *θ*_*MAX*_ = 0.31, *m* = 0.4}. (a) Spatial distribution of disease intensity at the equilibrium expressed as a linear interpolation of mean worm burden *W*_*i*_ at the aggregated network nodes (black dots) in log_10_-scale. (b) Spatial patterns of disease spread predicted by the rescaled dominant eigenvector **g_0_** of the system at the transcritical bifurcation of the DFE in log_10_-scale.

We first explore the spatial patterns of disease spread by mapping the components of the dominant eigenvector of the linearised system around the DFE. When compared to the equilibrium state of the system, [54] showed that in the case of micro-parasitic waterborne diseases characterized by fast dynamics, **g_0_** is an accurate predictor of the spatial distribution of cases both in the early phase of the outbreak and at the epidemic peak. In the perspective of mathematical modelling, schistosomiasis differs from cholera-like diseases in that it could be considered as a slow-fast dynamical system [83], meaning having rate parameters spanning 3 orders of magnitude (see Table 1). Indeed, by definition, the dominant eigenvector of the Jacobian of the linearised system approximates well the trajectory close to the DFE, but its predictive power of the endemic equilibrium remains to be verified. This is done by comparing the predicted spatial patterns of disease with the state of the system at the endemic equilibrium. The system at the national level has a prohibitively large number of state variables (4 × *n* ≈ 40′000) that hinders running the connected ordinary differential equations (ODE) system to endemic equilibrium using traditional numerical solvers on standard computing software. Specialized high-performance computing techniques could permit running the full system in real time—however, this beyond the scope of this work. To test the applicability of the eigenvector analysis to the full Burkina Faso settlement network we first perform the analysis on an aggregated network of 1′000 nodes to prove the relevance of the approach for slow-fast systems. The aggregated network is obtained by geographical clustering (using the *k-means* algorithm) of the full 10’592 node network. Spatial patterns of disease spread are obtained by rescaling the dominant eigenvector **g_0_** of the GRM to represent **W**, the mean worm burden along model trajectories diverging from the DFE. The results illustrated in Fig 3 show that at the transcritical bifurcation the rescaled dominant eigenvector reproduces well the spatial patterns obtained by plotting the worm burden components of the endemic equilibrium as simulated by Model 1 on the aggregated network. In particular, the disease hotspot is accurately predicted by the dominant eigenvector approach. Conversely, disease intensity levels in western parts of the country are slightly under-predicted by the rescaled dominant eigenvector **g_0_**. It is important to note that in the present context we define as a disease hotspot a geographical area characterized by mean worm burden in excess of 90% of the maximal simulated value, as obtained by numerical simulation of the aggregated system. Hotspots do not necessarily correspond to observed historical prevalence levels in the epidemiological data presented in Fig 1. These results suggest that the method holds for the macro-parasitic dynamical system representation of schistosomiasis transmission, at least close to the transcritical bifurcation. This result illustrate the usefulness of our approach in assessing the conditions for determining elimination thresholds, and their susceptibility to variations in transmission conditions.

#### Spatial patterns at the national level

**Fig 4:**
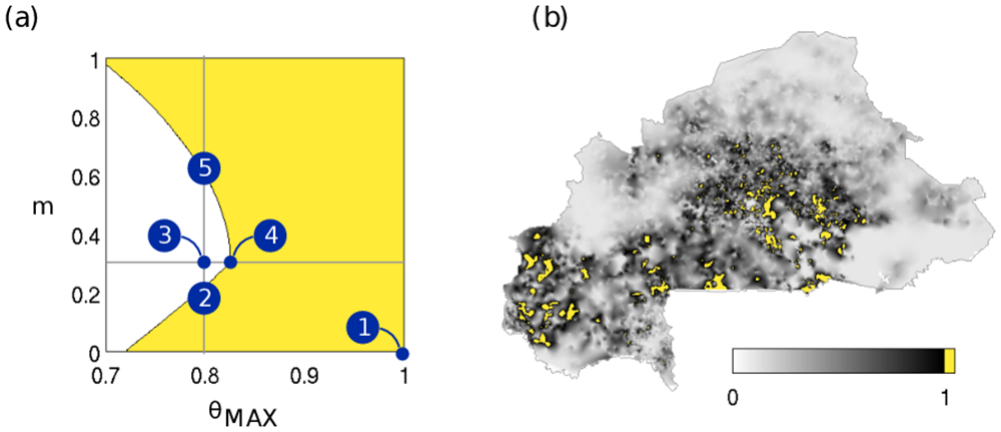
Pathogen invasion conditions and local transmission risk. (a) Stability diagram of the DFE in terms of contamination and exposure rates and the fraction of mobile people for intermediate urban contamination/exposure rates(*α* = 0.01). Points in the white[yellow] area represent parameter combinations yielding a stable[unstable] DFE. Parasite invasion occurs when crossing the bifurcation curve (black line, *g*_0_ = 1). (b) Local conditions of pathogen invasion risk in terms of *R*_0_ for no human mobility and maximal contact rates (point (1) in panel a). Areas presenting *R*_0,*i*_ > 1 (in yellow) experience sustained pathogen invasion and schistosomiasis is introduced in the node. Even for maximal contact rates locally schistosomiasis-prone areas are restrained to the south-western and central parts of the country.

**Fig 5:**
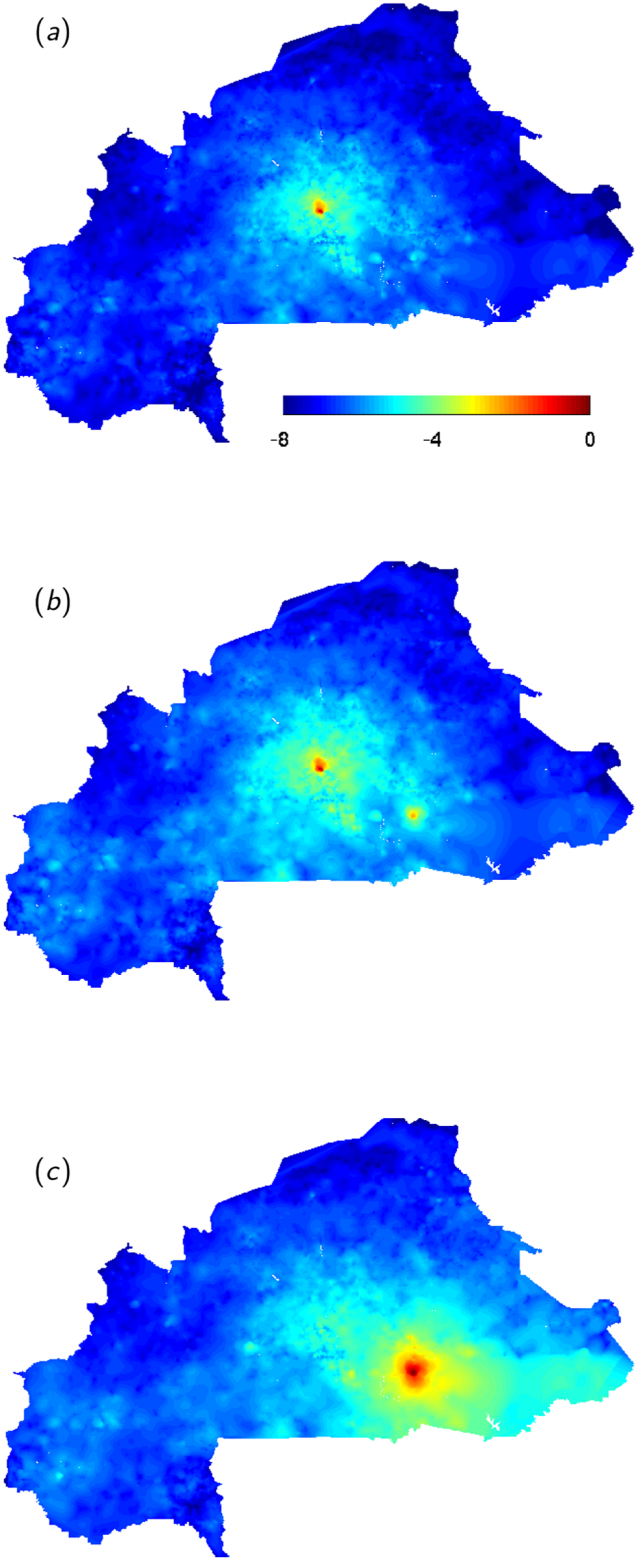
Human mobility and spatial patterns of the disease in the case of intermediate urban schistosomiasis. (a-c) Predicted log_10_-rescaled values of the mean worm burden compartment **W** for increasing levels of human mobility (*m* = 0.2, 0.3, 0.65) along the bifurcation line (points d-e-f in panel a) and the *θ*_*MAX*_ = 0.8 transect (points 2-4-5 in panel a).

The eigenvector approach allows the analysis of schistosomiasis spread on the full network of Burkina Faso. We chose to study spatial patterns for three mobility regimes along the *g*_0_ = 1 contour line in Fig 2 by tuning the free parameters (*m* and *θ*_*MAX*_) to place the system precisely at the transcritical bifurcation that determines the instability of the DFE, as illustrated in Fig 4. It is noteworthy that the dominant eigenvector analysis holds only close to the bifurcation which represents conditions tending towards elimination, thus relevant to surveillance-response strategies, as opposed to conditions of high endemicity characterized by *g*_0_ > > 1. Fig 5 illustrates the effect of connectivity on disease spread in comparison to local pathogen invasion condition, expressed by the local basic reproduction number *R*_0,*i*_ (panel (*b*) in Fig 4, corresponding to point (1) in panel (*a*)). Local potential for pathogen invasion results from the interaction between local population density and snail presence. At low human mobility intensity the disease concentrates around the populated areas in the center of the country (panel (*a*), point (2) in Fig 4(*a*)). By increasing mobility, the dilution effect causes the DFE to become stable and the pathogen cannot invade the country (point (3) in Fig 4(*a*)). For parameter combinations yielding pathogen invasion at intermediate human mobility (*m* = 0.3), the spatial patterns of disease spread shift towards the South-East with the appearance of a second hotspot in an area with favourable local conditions (panel (*b*), point (4) in Fig 4(*a*)). The effect of mobility is most visible when the majority of people are mobile (*m* = 0.65). Indeed, the South-Eastern hotspot takes over the central one and the disease is predicted to spread to the whole South-Eastern part of the country in areas where the local reproduction number is below 1, indicating unfavourable local conditions for transmission (panel (*c*), point (5) in Fig 4(*a*)). It is interesting to note that the spatial patterns of disease spread on the aggregated network reproduce reasonably well the ones observed on the full network, thus supporting our assumptions. The capacity to predict the spatial patterns of the disease close to the disease free equilibrium could provide a tool for prioritizing surveillance sampling in those areas where schistosomiasis is predicted to pickup the most, thus were the re-invasion signal in the mean worm burden would be the strongest.

### Water resources development and human mobility

**Fig 6:**
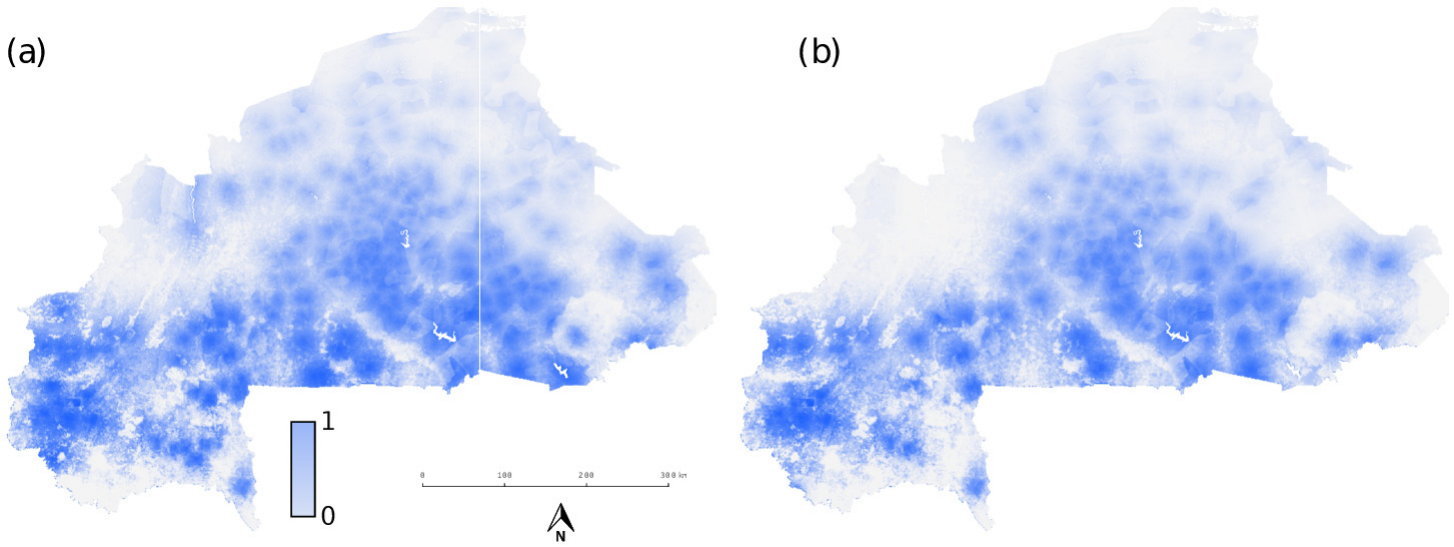
Impact of water resources on the ecological range of the intermediate host. (a) *B. pfeifferi* probability of presence for the current scenario of water resources development. (b) Probability of presence for an example of the alternative scenario with half the number of reservoirs (*ϵ* = 0.5). The predicted species distribution of *B. pfeifferi* by the MaxEnt approach reveals the importance of distance to water surfaces along with the influence of the North-South precipitation/temperature gradient which prevents the snail to colonize the Northern parts of the territory (see S1 Text for more details). The example of one realization of the scenario with half the reservoirs illustrates the reduction of the ecological range of the intermediate host, necessary but not sufficient condition for explaining the observed results.

**Fig 7:**
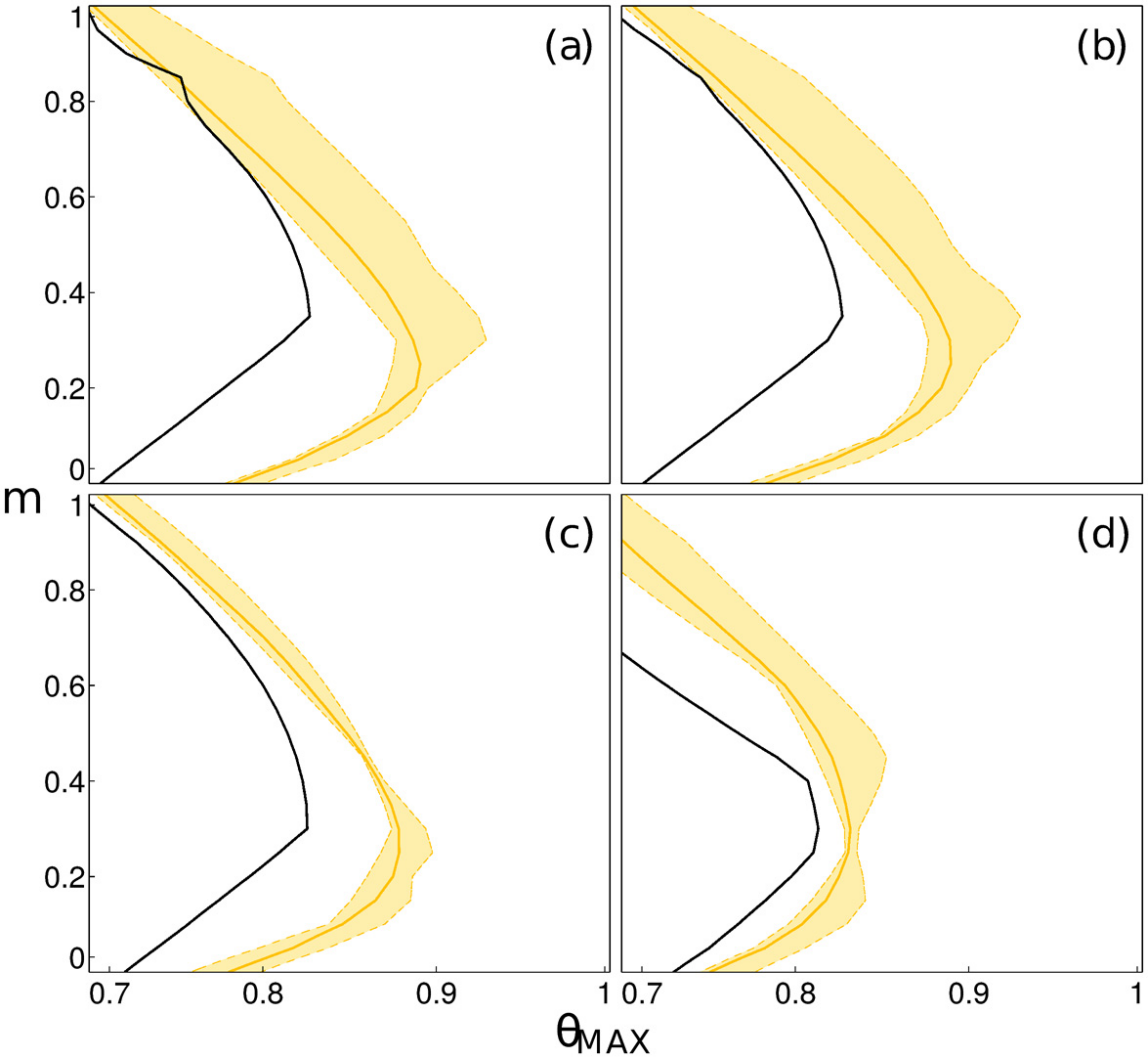
Investigation of the side-effects of water resources development in terms of pathogen invasion conditions of schistosomiasis. The bifurcation curves (*g*_0_ = 1) of the DFE are plotted for a level of water resources development corresponding to 50% of the existing reservoirs, and for increasing values of *α* ∈ {0.0001, 0.001, 0.01, 0.1} (respectively in panels (a) to (d)). Stability plots are given as a function of the maximal contamination and exposure rate parameter *θ*_*MAX*_ and the fraction of mobile people *m*. Yellow curves represent parasite invasion conditions for the alternative scenarios of water resources development, while black lines refer to the current situation (same as Fig 2), and are reported here for reference. Colour shadings represent 95% confidence intervals based on 10 scenario realizations by random removal of existing reservoirs. Regions to the right of the black and coloured lines correspond to conditions of pathogen invasion of the country, i.e. an unstable DFE, for the current and alternative scenarios respectively.

Water resources development directly impacts large-scale schistosomiasis transmission by altering the probability of presence of the intermediate snail host in the environment. This is explicitly accounted for in our approach through the distance to water, $D_{water}$, of a human settlement. To investigate the side-effects of water resources development on schistosomiasis in the country, *B. pfeifferi* probability of presence was re-evaluated using MaxEnt for alternative scenarios of water resources development by randomly removing existing reservoirs to consider different numbers of built dams expressed as a fraction *ϵ* of the existing ones, and the stability of the DFE recomputed for parameter combinations used in Fig 2. The evolution of water resources development in Burkina Faso (a 5-fold increase in the number of reservoirs in the past 60 years [46]) was explored by varying *ϵ* in the set {0.25, 0.5, 0.75} of existing reservoirs, which represents increasing numbers of built reservoirs. An illustration of the predicted changes in the ecological range of the intermediate host due to water resources development, in comparison with the current scenario, is given in Fig 6. Uncertainty in the stability predictions induced by random removal of reservoirs was assessed by repeating the procedure 10 times for each value of *ϵ* and taking the 95% confidence intervals of the realizations of the resulting stability lines. The effect of water resources development on pathogen invasion is illustrated in Fig 7 for the case of *ϵ* = 0.5, results for the other two scenarios are similar and reported in S5 Fig. For all the reduced water resources development scenarios, the DFE tends to be stable for a wider set of mobility and contact parameters, for all levels of urban contamination and exposure rates. Examining the stability diagrams in Fig 7, it becomes evident that human mobility plays an important role in the stability of the DFE, thus of pathogen invasion conditions, in these alternative scenarios. Indeed the interplay between human mobility and water resources development is greatly conditioned by the level of urban contamination/exposure rates. For *α* < 0.1 the effect of building dams is most detrimental for low levels of human mobility (*m* < 0.25), while the bifurcation lines tend to be close to the invasion condition of the current scenario for *m* > 0.8. On the other hand, for high levels of urban transmission of schistosomiasis (*α* = 0.1) human mobility accentuates the negative impact of the increasing number of dams by augmenting the gap between the bifurcations curves of the alternative and current scenarios *m* > 0.6. Furthermore, uncertainty in the stability of the DFE increases with the fraction of mobile people for all *α*’s. These observations can be seen as the contribution of less connected settlements, where contamination and exposure are high, to the overall (in)stability condition of the DFE. By increasing urban contamination and exposure rates, nodes with high connectivity also present high transmission, thus having a large impact on the stability of the system. When removing reservoirs that determine the number of snails in these key nodes, the stability region of the DFE expands. In other words, the system is more dependent on a small set of highly connected nodes, thus the construction of dams close to these points has a much larger effect on stability. The uncertainty associated to the location of the stability line is thus directly related to the random removal of the reservoirs influencing snail abundance in these key nodes. Human mobility therefore exacerbates the effect of water resources development on risk of pathogen invasion in the case of high urban contamination and exposure rates.

Although preliminary, these results illustrate the use of the proposed eigenvector analysis of the GRM to quantify the potential impacts of water resources development in a spatially explicit framework that includes connectivity mechanisms such as human mobility. In particular, we concur with previous studies suggesting that strategies to mitigate negative effects on human health should become integral parts in the planning, implementation, and operation of future water resources development projects [44]. In particular the interplay between the water resources development and connectivity induced by human mobility are deemed to be important in the risk of re-emergence of the disease if elimination is attained.

### A roadmap for Burkina Faso

Although the main focus of this work is on the system conditions requiring deployment of intervention measures in the perspective of elimination, thus far from the endemic equilibrium, an appropriate calibration would need to be validated against historical data for the specific context of Burkina Faso. The first step in this direction would require the availability of comprehensive historical and national scale data on disease prevalence and infection intensity currently unavailable in a standardized accessible form. These data would also allow quantification of the associated uncertainty of the predictions.

The second step could consist in the relaxation of simplifying assumptions of the model by [12]. Namely the assumptions are: (1) constant, although spatially heterogeneous, human and (2) snail populations, (3) population-dependent contamination and exposure rates, (4) no hydrologic transport of parasite larvae (5) nor of the snail intermediate host, and finally (6) mobility patterns modulated by population density. To that end, Burkina Faso seems uniquely positioned in Africa for capitalizing on existing field and epidemiological data [21, 27]. Here, parameter values were taken from previously investigated configurations, but field data collection would be needed to strengthen the use of the results presented here. If assumptions (1) and (4) are deemed to be reasonable for studying schistosomiasis at the country-level, the remaining points still need to be addressed.

Snail population dynamics (2) needs to be explicitly modelled with respect to local environmental conditions governing its habitat and its population dynamics, mainly driven by hydrology [28, 84, 85]. Furthermore, the seasonal variation of eco-hydrologic parameters would need to be properly addressed and incorporated in the modelling framework [55]. For both of these aspects of particular use would be local hydro-climatic data at suitable temporal and spatial resolutions in Burkina Faso, to be assimilated along with remotely sensed data with of bridging the existing gaps in their use for schistosomiasis risk profiling [86].

Contact and contamination rates (3) strongly depend on human-water contacts driven by local socio-economic realities [32, 47, 87], and are of particular importance to quantify the success of control measures through education and access to clean water, sanitation and hygiene (WASH). A generalizable framework would need to be developed for the specific context of Burkina Faso where detailed geo-referenced data are increasingly available especially on improved water sources, sanitation infrastructure and natural or man-made waterbodies, complemented by surveys and participatory-observant methods in distinct climatic, water resource availability and socio-economic contexts [31, 32]. This would reduce the uncertainty introduced with the population-density based approach presented here for estimating water-contact rates, with a particular focus on the seasonal dynamics of water contacts across the territory.

Hydrologic transport of the intermediate host (4) regarding transport rates, variability, snail species specificities and seasonality is also in need of an assessment regarding its possible inclusion in large-scale modelling frameworks.

Finally, increasing access to human mobility data such as cell phone records would not only remove the necessity for modelling mobility patterns (6) [75, 88], but would also permit the validation of existing mobility models that are generally based on data from highly motorized countries [74, 76] against the specific mobility characteristics of Sub-Saharan Africa for their use in the context of schistosomiasis study. Indeed we note that other mobility models such as the gravity model imply different underlying assumptions. A detailed study of the relative merits of radiation versus gravity-like models has been the subject of recent attention. A relevant point for the work we present here is that the radiation model was found to systematically under-estimate long-range mobility fluxes, whereas the gravity model overestimated them [89]. In other words, the radiation model produces conservative estimates of mobility with respect to a gravity model and potentially to real mobility patterns. This implies that our conclusions are a lower bound on the effects of mobility on schistosomiasis transmission in the country. Nevertheless workers reported in the same study that both models failed to capture short-range mobility in rural areas, which could be of great importance for local schistosomiasis transmission. In light of these considerations the radiation model was chosen to investigate the impact of human mobility on schistosomiasis in Burkina Faso at this stage. Needless to say, the availability of real mobility data would overcome the need for modelling, as observed mobility fluxes could be used directly (see e.g. [74]), and would permit a thorough assessment of existing mobility models in the context of Burkina Faso. In fact, the mobility matrix *Q*_*ij*_ and the mobile fraction *m*_*i*_ of the *H*_*i*_ inhabitants in each location could be inserted directly into our mathematical framework.

### Conclusions

By implementing dynamical system analysis techniques recently developed for waterborne diseases, we have highlighted the roles of human mobility and water resources development in the spread of intestinal schistosomiasis in Burkina Faso. Human mobility was shown to play a role not only in the degree of success of invasion of the network of human settlements by the pathogen, but also in the spatial patterns of disease spread. For small fractions of mobile people mobility induced a dilution effect of the parasite concentration in accessible waters leading to an effective prevention of parasite invasions, in agreement with previous results derived at much smaller spatial scales. Even in settings unfavourable to pathogen invasion, human mobility had a large effect on the time delay of transmission interruption. Above a threshold value, mobility prompted a systematic exacerbation of the disease by shifting its hotspots from the most populated areas (even where local conditions warrant local reproduction numbers larger than one) to more transmission-prone areas with higher contamination and exposure rates and snail abundance.

Predicting the spread of the disease based on water resources development was also investigated, specifically by quantifying the modification of the stability conditions of the disease-free equilibrium resulting from anthropogenic expansions of suitable habitats for *B. pfeifferi*, the snail acting as intermediate host in the life cycle of the parasite *S. mansoni*. By considering theoretical scenarios representative of pre-existing levels of water resources development in the country, we showed that the marked increase in the number of dams prompted by water development projects favoured pathogen invasion. For low urban schistosomiasis transmission, the greatest effects of water resources development were found to occur for low levels of human mobility, thus favouring localized transmission which maximizes the effect of each additional dam built. Interestingly, intense human mobility was also shown to exacerbate the impact of the building of the dams in the case of high levels of urban contamination and exposure rates.

We conclude that insight from the tools we develop here could directly inform national and regional control measures in the perspective of elimination in terms of pathogen invasion conditions and initial spatial spread, thus contributing to two of the points brought to the fore as research priorities for helminth modelling efforts [57]. Both prevention and intervention at regional scales, in fact, need to address the central role of human mobility in the perspective of the deployment of surveillance-response mechanisms and the localization of treatment measures. Controls of urban contamination and exposure rates, say by reducing contamination rates by control programs like WASH, could be measured against predicted disease burden reductions. Even at the current state of development, results of management alternatives obtained from mathematical models are potentially informative for epidemiological decisions in conditions of close-to-threshold transmission that will hopefully be the rule in the near future thanks to elimination programs in sub-Saharan Africa and Burkina Faso. The proposed theoretical framework provides opportunities for the design of control indicators based on stability, possibly also taking into account the timescales of pathogen extinction. Overall, our work supports the key role that mathematical toolboxes built on spatially explicit models of disease dynamics play for the prediction of the spatial patterns of schistosomiasis at regional scales.

## Supporting Information

S1 Text Generalized reproduction matrix and dominant eigenvector derivation of the spatially explicit schistosomiasis transmission model. Derivation of stability conditions of the connected space.
(PDF)

S1 Table Spatial data sources. Data sources, description and download links.
(PDF)

S1 Fig Population distribution in Burkina Faso and Voronoi tessalation used for building the network. Population density is given at a resolution of ≈ 1km^2^. Permission to publish granted from East View Information Services/LandScan.
(TIF)

S2 Fig Covariates used in the species distribution modelling of the snail intermediate host using MaxEnt. All data sources are analogous to those used in [72].
(TIF)

S3 Fig Jacknife analysis of covariate importance. Results are shown for one fold of the fitting procedure. Importance is measured in terms of the regularized training gain of the MaxEnt model when removing a given variable (light blue) and using only that variable (dark blue). The former expresses the information contained in the given covariate that is different from other variables. The latter the specific explanatory power contained in the variable. The results show that distance to reservoirs is both the covariate with the largest explanatory power, and with the highest information content not contained in the other covariates.
(TIF)

S4 Fig Sigmoid functional forms for exposure/contamination. The curves are given here for *θ*_*MAX*_ = 1 as a function of population in node *i*, *H*_*i*_, and for values of *α* used in model exploration.
(TIF)

S5 Fig Water resources development in terms of pathogen invasion conditions of schistosomiasis. The bifurcation curves (*g*_0_ = 1) of the DFE are plotted for different levels of water resources development expressed in terms of fraction of the existing reservoirs. Stability plots are given as a function of the maximal contact/exposure rate parameter *θ*_*MAX*_ and the fraction of mobile people *m*. Coloured (green, red) curves represent parasite invasion conditions for the alternative scenarios of water resources development, while black lines refer to the current situation (same as Fig 2 in main text), and are reported here for reference. Colour shadings represent 95% confidence intervals based on 10 scenario realizations by random removal of existing reservoirs. Regions to the right of the black and coloured lines correspond to conditions of pathogen invasion of the country, i.e. an unstable DFE, for the current and alternative scenarios respectively.
(TIF)
