# Supplementary material for: A Theoretical Analysis of the Geography of Schistosomiasis in Burkina Faso Highlights the Roles of Human Mobility and Water Resources Development in Disease Transmission
Source: PLoS Negl Trop Dis. 2015 Oct 29;9(10):e0004127. doi: 10.1371/journal.pntd.0004127 (PMC4625963; doi:10.1371/journal.pntd.0004127)
Supplement: S1 Text — Derivation of stability conditions of the connected space. (PDF) [file pntd.0004127.s001.pdf]

Derivation of the stability criteria for the connected schistosomiasis model

1 The local model

Let us start with the analysis of the parasite invasion condition in MacDonald's (1965) spatially implicit model. We recall that the model can be written as

$$
\begin{aligned}
\frac{dW}{dt} &= a\theta C - \gamma W \\
\frac{dY}{dt} &= bM(1-Y) - \nu Y \\
\frac{dC}{dt} &= \frac{\Pi_C}{V} NY - \mu_C C \\
\frac{dM}{dt} &= \frac{\Pi_M}{V} \theta' H \frac{W}{2} - \mu_M M \ .
\end{aligned}
$$

As the model is a positive system (namely its state variables can never become negative if the system is initialized at generic non-negative conditions), the bifurcation from stable to unstable of the disease-free equilibrium (DFE) $\mathbf{X_0} = [0, 0, 0, 0]^T$, i.e. a state of the system characterized by the absence of the parasite, can only occur via an exchange of stability. This implies that the DFE switches from being a stable equilibrium to being a saddle (i.e. an equilibrium with one unstable manifold) through a so-called transcritical bifurcation [**?**]. The condition for the bifurcation to occur (hence for parasite invasion) is thus determined by the stability properties of the Jacobian of the system evaluated at the DFE, i.e.

$$
\mathbf{J_0} = \begin{bmatrix} -\gamma & 0 & a\theta & 0 \\ 0 & -\nu & 0 & b \\ 0 & \frac{\Pi_C}{V} N & -\mu_C & 0 \\ \frac{\Pi_M}{V}\theta' \frac{H}{2} & 0 & 0 & -\mu \end{bmatrix} .
$$

Specifically, the DFE is asymptotically stable (thus precluding parasite invasion) if and only if the dominant eigenvalue of $\mathbf{J_0}$ is strictly negative. In this case the determinant of $\mathbf{J_0}$ is positive, because the Jacobian is a matrix of even order. The change of stability for $\mathbf{X_0}$ is thus obviously associated to the condition $\det(\mathbf{J_0}) = 0$, i.e.

$$\det(\mathbf{J_0}) = -\gamma \det\left(\begin{bmatrix} -\nu & 0 & b \\ \frac{\Pi_C}{V}N & -\mu_C & 0 \\ 0 & 0 & -\mu_M \end{bmatrix}\right) - a\theta \det\left(\begin{bmatrix} 0 & -\nu & b \\ 0 & \frac{\Pi_C}{V}N & 0 \\ \frac{\Pi_M}{V}\theta'\frac{H}{2} & 0 & -\mu_M \end{bmatrix}\right) =$$
$$= -\gamma\nu\mu_C\mu_M + \frac{ab\theta\theta'\Pi_c\Pi_M HN}{2V^2} = 0 \,.$$

The previous condition can be equivalently written in terms of the basic reproduction number R_0 as

$$R_0 = \frac{ab\theta\theta'\Pi_C\Pi_M HN}{2\gamma\nu\mu_C\mu_M V^2} = 1 \,,$$

and the parasite can invade a disease-free community if and only if $R_0 > 1$.

2 The spatially explicit network model

A spatially explicit version of Macdonald's model, accounting for human mobility and hydrological transport of the intermediate larval stages of the parasite (and neglecting snail mobility) proposed by [**?**] is formulated as follows:

$$\frac{dW_i}{dt} = a\left[(1-m_i)\theta_i C_i + m_i \sum_{j=1}^{n} Q_{ij}\theta_j C_j\right] - \gamma W_i$$
$$\frac{dY_i}{dt} = bM_i(1-Y_i) - \nu Y_i$$
$$\frac{dC_i}{dt} = \frac{\Pi_C}{V_i}N_i Y_i - \mu_C C_i - l_i^C C_i + \sum_{j=1}^{n} l_j^C P_{ji} S_{ji}^C \frac{V_j}{V_i} C_j$$
$$\frac{dM_i}{dt} = \frac{\Pi_M}{V}\theta_i'\left[(1-m_i)H_i\frac{W_i}{2} + \sum_{j=1}^{n} m_j H_j \frac{W_j}{2} Q_{ji}\right] - \mu_M M_i - l_i^M M_i + \sum_{j=1}^{n} l_j^M P_{ji} S_{ji}^M \frac{V_j}{V_i} M_j \,.$$

Analogously to the spatially implicit case, the condition for parasite invasion in the network model is determined by the stability properties of the Jacobian matrix of the system linearised at the disease-free

equilibrium. Switching to matrix notation, we have

$$\mathbf{J_0^*} = \begin{bmatrix} \boldsymbol{\mathcal{A}} & \boldsymbol{\mathcal{B}} \\ \boldsymbol{\mathcal{C}} & \boldsymbol{\mathcal{D}} \end{bmatrix},$$

where

$$\boldsymbol{\mathcal{A}} = \begin{bmatrix} -\gamma\mathbf{I} & 0 \\ 0 & -\nu\mathbf{I} \end{bmatrix} \qquad \boldsymbol{\mathcal{B}} = \begin{bmatrix} a(\mathbf{I}-\mathbf{m}+\mathbf{mQ})\boldsymbol{\theta} & 0 \\ 0 & b\mathbf{I} \end{bmatrix}$$

$$\boldsymbol{\mathcal{C}} = \begin{bmatrix} 0 & \Pi_C\mathbf{V}^{-1}\mathbf{N} \\ \frac{\Pi_M}{2}\mathbf{V}^{-1}\boldsymbol{\theta}'(\mathbf{I}-\mathbf{m}+\mathbf{Q}^T\mathbf{m})\mathbf{H} & 0 \end{bmatrix} \qquad \boldsymbol{\mathcal{D}} = \begin{bmatrix} -\mu_C\mathbf{I}+\mathbf{T_C} & 0 \\ 0 & -\mu_M\mathbf{I}+\mathbf{T_M} \end{bmatrix}.$$

In the previous expressions $\mathbf{I}$ is the identity matrix; $\mathbf{m}$, $\boldsymbol{\theta}$, $\mathbf{V}$, $\mathbf{N}$, $\boldsymbol{\theta}'$ and $\mathbf{H}$ are diagonal matrices whose non-zero elements are made up by the parameters m_i, θ_i, V_i, N_i, θ_i' and H_i, respectively; $\mathbf{Q} = [Q_{ij}]$ is the connectivity matrix for human mobility; $\mathbf{T_C} = \left(\mathbf{V}^{-1}\mathbf{P_C}^T\mathbf{V}-\mathbf{I}\right)\mathbf{l_C}$ and $\mathbf{T_M} = \left(\mathbf{V}^{-1}\mathbf{P_M}^T\mathbf{V}-\mathbf{I}\right)\mathbf{l_M}$, where $\mathbf{P_C} = [P_{ij}S_{ij}^C] = \mathbf{P}\circ\mathbf{S_C}$ and $\mathbf{P_M} = [P_{ij}S_{ij}^M] = \mathbf{P}\circ\mathbf{S_M}$ are transport matrices accounting for hydrological connectivity and larval survival during transport, and $\mathbf{l_C}$ and $\mathbf{l_M}$ are diagonal matrices whose non-zero elements are the local values of l_i^C and l_i^M, respectively.

Let us preliminary note that the off-diagonal entries of $\mathbf{J_0^*}$ are all nonnegative and at least one diagonal entry is negative, thus $\mathbf{J_0^*}$ is a proper Metzler matrix [?] and its eigenvalue with maximal real part (dominant eigenvalue) is real. If the union of graphs associated with matrices $\mathbf{P}$ and $\mathbf{Q}$ is strongly connected, then the graph associated with $\mathbf{J_0^*}$ is also strongly connected. Therefore we can apply Perron-Frobenius theorem for irreducible matrices [?] and state that the dominant eigenvalue of $\mathbf{J_0^*}$ is the maximum simple real root of the characteristic polynomial. The condition for the transcritical bifurcation of the DFE is that the dominant eigenvalue crosses the imaginary axis at zero, namely that the determinant of $\mathbf{J_0^*}$ is zero [?]. When the DFE is stable (sufficient condition to prevent parasite invasion), all the eigenvalues of $\mathbf{J_0^*}$ have negative real parts and $\det(\mathbf{J_0^*})$ is positive because $\mathbf{J_0^*}$ is a matrix of order $4n$. The DFE becomes unstable (necessary condition for parasite invasion) when $\det(\mathbf{J_0^*})$ switches from positive to negative, or equivalently when the dominant eigenvalue of $\mathbf{J_0^*}$ switches from negative to positive.

Noting the block structure of $\mathbf{J_0^*}$ and that $\boldsymbol{\mathcal{A}}$ is a piece-wise scalar matrix (thus $\boldsymbol{\mathcal{A}}\boldsymbol{\mathcal{B}} = \boldsymbol{\mathcal{B}}\boldsymbol{\mathcal{A}}$), the

determinant of $\mathbf{J_0^*}$ can be computed as [?]

$$\det(\mathbf{J_0^*}) = \det(\boldsymbol{\mathcal{D}}\boldsymbol{\mathcal{A}} - \boldsymbol{\mathcal{C}}\boldsymbol{\mathcal{B}}) =$$

$$= \det\left(\begin{bmatrix} \gamma(\mu_C\mathbf{I} - \mathbf{T_C}) & -b\Pi_C\mathbf{V}^{-1}\mathbf{N} \\ -\frac{a\Pi_M}{2}\mathbf{V}^{-1}\boldsymbol{\theta}'(\mathbf{I} - \mathbf{m} + \mathbf{Q}^T\mathbf{m})\mathbf{H}(\mathbf{I} - \mathbf{m} + \mathbf{m}\mathbf{Q})\boldsymbol{\theta} & \nu(\mu_M\mathbf{I} - \mathbf{T_M}) \end{bmatrix}\right) = \det(\mathbf{J_0^{**}})\ .$$

Writing $\mathbf{J_0^{**}}$ as

$$\mathbf{J_0^{**}} = \begin{bmatrix} \boldsymbol{\mathcal{U}} & \boldsymbol{\mathcal{W}} \\ \boldsymbol{\mathcal{X}} & \boldsymbol{\mathcal{Z}} \end{bmatrix}$$

it is possible to show [see again ?] that

$$\det(\mathbf{J_0^{**}}) = \det(\boldsymbol{\mathcal{U}}\boldsymbol{\mathcal{Z}} - \boldsymbol{\mathcal{W}}\boldsymbol{\mathcal{Z}}^{-1}\boldsymbol{\mathcal{X}}\boldsymbol{\mathcal{Z}})\ ,$$

obviously provided that matrix $\boldsymbol{\mathcal{Z}}$ is invertible. With straightforward algebraic manipulations the right-hand side of the previous equation can be written in a form that is more amenable to further analysis, i.e.

$$\det(\boldsymbol{\mathcal{U}}\boldsymbol{\mathcal{Z}} - \boldsymbol{\mathcal{W}}\boldsymbol{\mathcal{Z}}^{-1}\boldsymbol{\mathcal{X}}\boldsymbol{\mathcal{Z}}) = \det(\boldsymbol{\mathcal{W}})\det(\boldsymbol{\mathcal{Z}}\boldsymbol{\mathcal{W}}^{-1}\boldsymbol{\mathcal{U}} - \boldsymbol{\mathcal{X}}) = \det(\boldsymbol{\mathcal{W}})\det(\mathbf{J_0^{***}})\ ,$$

which additionally requires matrix $\boldsymbol{\mathcal{W}}$ to be invertible. If the conditions on matrices $\boldsymbol{\mathcal{Z}}$ and $\boldsymbol{\mathcal{W}}$ are verified, the bifurcation condition $\det(\mathbf{J_0^*}) = 0$ corresponds to $\det(\mathbf{J_0^{***}}) = 0$. The determinant of $\mathbf{J_0^{***}}$ can be written as

$$\begin{aligned}
&\det(\mathbf{J_0^{***}}) = \\
&\quad = \det\left(\nu(\mu_M\mathbf{I} - \mathbf{T_M})\left(-\frac{1}{b\Pi_C}\mathbf{V}\mathbf{N}^{-1}\right)\gamma(\mu_C\mathbf{I} - \mathbf{T_C}) + \right.\\
&\quad \left. + \frac{a\Pi_M}{2}\mathbf{V}^{-1}\boldsymbol{\theta}'(\mathbf{I} - \mathbf{m} + \mathbf{Q}^T\mathbf{m})\mathbf{H}(\mathbf{I} - \mathbf{m} + \mathbf{m}\mathbf{Q})\boldsymbol{\theta}\right) = \\
&\quad = \det\left(\frac{a\Pi_M}{2}\mathbf{V}^{-1}\boldsymbol{\theta}'(\mathbf{I} - \mathbf{m} + \mathbf{Q}^T\mathbf{m})\mathbf{H}(\mathbf{I} - \mathbf{m} + \mathbf{m}\mathbf{Q})\boldsymbol{\theta} - \frac{\gamma\nu}{b\Pi_C}(\mu_M\mathbf{I} - \mathbf{T_M})\mathbf{V}\mathbf{N}^{-1}(\mu_C\mathbf{I} - \mathbf{T_C})\right) = \\
&\quad = \det\left(\frac{ab\Pi_C\Pi_M}{2\gamma\nu\mu_C\mu_M}\mathbf{N}\mathbf{V}^{-1}\boldsymbol{\theta}'[(\mathbf{I} - \mathbf{m})\mathbf{H}(\mathbf{I} - \mathbf{m}) + (\mathbf{I} - \mathbf{m})\mathbf{H}\mathbf{m}\mathbf{Q} + \mathbf{Q}^T\mathbf{m}\mathbf{H}(\mathbf{I} - \mathbf{m}) + \mathbf{Q}^T\mathbf{m}\mathbf{H}\mathbf{m}\mathbf{Q}]\boldsymbol{\theta}\mathbf{V}^{-1} + \right.\\
&\quad \left. - \frac{1}{\mu_C\mu_M}\mathbf{N}(\mu_M\mathbf{I} - \mathbf{T_M})\mathbf{V}\mathbf{N}^{-1}(\mu_C\mathbf{I} - \mathbf{T_C})\mathbf{V}^{-1}\right)\left(\frac{b\Pi_C}{\gamma\nu\mu_C\mu_M}\right)^n \det\left(\mathbf{N}^{-1}\right)\det(\mathbf{V}) = \\
&\quad = \det(\mathbf{J_0^{****}})\left(\frac{\gamma\nu\mu_C\mu_M}{b\Pi_C}\right)^n \det\left(\mathbf{N}^{-1}\right)\det(\mathbf{V})\ .
\end{aligned}$$

The condition $\det(\mathbf{J_0^{***}}) = 0$ is thus clearly equivalent to $\det(\mathbf{J_0^{****}}) = 0$, where

$$\begin{aligned}
&\det(\mathbf{J_0^{****}}) = \\
&= \det\left(\frac{ab\Pi_C\Pi_M}{2\gamma\nu\mu_C\mu_M}\mathbf{N}\left(\mathbf{V}^{-1}\right)^2 \boldsymbol{\theta'}(\mathbf{I}-\mathbf{m})^2\mathbf{H}\boldsymbol{\theta} + \right.\\
&+ \frac{ab\Pi_C\Pi_M}{2\gamma\nu\mu_C\mu_M}\mathbf{N}\mathbf{V}^{-1}\boldsymbol{\theta'}\left[(\mathbf{I}-\mathbf{m})\mathbf{m}\mathbf{H}\mathbf{Q} + \mathbf{Q}^T\mathbf{H}\mathbf{m}(\mathbf{I}-\mathbf{m}) + \mathbf{Q}^T\mathbf{m}^2\mathbf{H}\mathbf{Q}\right]\boldsymbol{\theta}\mathbf{V}^{-1} + \\
&\left. - \mathbf{N}\left(\mathbf{I} - \frac{1}{\mu_M}\mathbf{T_M}\right)\mathbf{V}\mathbf{N}^{-1}\left(\mathbf{I} - \frac{1}{\mu_C}\mathbf{T_C}\right)\mathbf{V}^{-1}\right) = \\
&= \det\left(\frac{ab\Pi_C\Pi_M}{2\gamma\nu\mu_C\mu_M}\mathbf{N}\left(\mathbf{V}^{-1}\right)^2 \boldsymbol{\theta'}(\mathbf{I}-\mathbf{m})^2\mathbf{H}\boldsymbol{\theta} + \right.\\
&+ \frac{ab\Pi_C\Pi_M}{2\gamma\nu\mu_C\mu_M}\mathbf{N}\mathbf{V}^{-1}\boldsymbol{\theta'}\left[(\mathbf{I}-\mathbf{m})\mathbf{m}\mathbf{H}\mathbf{Q} + \mathbf{Q}^T\mathbf{H}\mathbf{m}(\mathbf{I}-\mathbf{m}) + \mathbf{Q}^T\mathbf{m}^2\mathbf{H}\mathbf{Q}\right]\boldsymbol{\theta}\mathbf{V}^{-1} + \\
&\left. - \mathbf{I} + \frac{1}{\mu_C}\mathbf{V}\mathbf{T_C}\mathbf{V}^{-1} + \frac{1}{\mu_M}\mathbf{N}\mathbf{T_M}\mathbf{N}^{-1} - \frac{1}{\mu_C\mu_M}\mathbf{N}\mathbf{T_M}\mathbf{V}\mathbf{N}^{-1}\mathbf{T_C}\mathbf{V}^{-1}\right) .
\end{aligned}$$

If we introduce a diagonal matrix $\mathbf{R_0}$, whose non-zero elements are the local values R_{0i} of the basic reproduction number, i.e.

$$R_{0i} = \frac{ab\theta_i\theta_i'\Pi_C\Pi_M H_i N_i}{2\gamma\nu\mu_C\mu_M V_i^2} ,$$

a matrix

$$\mathbf{R_0^M}(\mathbf{m},\mathbf{Q}) = \frac{ab\Pi_C\Pi_M}{2\gamma\nu\mu_C\mu_M}\mathbf{N}\mathbf{V}^{-1}\boldsymbol{\theta'}\left[(\mathbf{I}-\mathbf{m})\mathbf{m}\mathbf{H}\mathbf{Q} + \mathbf{Q}^T\mathbf{H}\mathbf{m}(\mathbf{I}-\mathbf{m}) + \mathbf{Q}^T\mathbf{m}^2\mathbf{H}\mathbf{Q}\right]\boldsymbol{\theta}\mathbf{V}^{-1}$$

accounting for the effects of human mobility on disease transmission, and another matrix

$$\mathbf{T}(\mu_C, \mathbf{T_C}, \mu_M, \mathbf{T_M}) = \frac{1}{\mu_C}\mathbf{V}\mathbf{T_C}\mathbf{V}^{-1} + \frac{1}{\mu_M}\mathbf{N}\mathbf{T_M}\mathbf{N}^{-1} - \frac{1}{\mu_C\mu_M}\mathbf{N}\mathbf{T_M}\mathbf{V}\mathbf{N}^{-1}\mathbf{T_C}\mathbf{V}^{-1}$$

describing the effects of pathogen hydrological transport, it is possible to define a generalized reproduction matrix

$$\mathbf{G_0} = (\mathbf{I}-\mathbf{m})^2\mathbf{R_0} + \mathbf{R_0^M}(\mathbf{m},\mathbf{Q}) + \mathbf{T}(\mu_C, \mathbf{T_C}, \mu_M, \mathbf{T_M}) .$$

The original bifurcation condition $\det(\mathbf{J_0^*}) = 0$ can thus be written as

$$\det\left(\mathbf{I} - \mathbf{G_0}\right) = 0 .$$

Let λ_i ($i = 1..n$) be the eigenvalues of $\mathbf{G_0}$ and $g_0 = \max_i(\lambda_i)$. Then

$$\det(\mathbf{I} - \mathbf{G_0}) = \prod_{i=1}^{n}(1 - \lambda_i) \; .$$

Therefore, the determinant of $\mathbf{J_0^*}$ switches from positive to negative when g_0, the dominant eigenvalue of $\mathbf{G_0}$, switches from being smaller to being larger than one. It follows that the condition for the instability of the DFE (hence for parasite invasion) is $g_0 > 1$.

We note that in the absence of hydrological dispersal ($l_i^C = l_i^M = 0$ for all i) and human mobility ($m_i = 0$ for all i), i.e. in the case of completely isolated communities, the condition for parasite invasion becomes

$$\det(\mathbf{I} - \mathbf{R_0}) = 0 \; ,$$

which is obviously satisfied when the largest of the R_{0i} values is equal to one – which corresponds to the classical criterion obtained in a spatially implicit context. However, the introduction of human mobility and hydrological connections makes the condition $g_0 > 1$ for parasite invasion absolutely nontrivial. This criterion synthesizes the intertwined effects of local epidemiological processes, human mobility and hydrological transport on parasite invasion. As a matter of fact, $\mathbf{G_0}$ is the sum of three matrices: one depending on local dynamics only, the other two (non-linearly) on spatial coupling mechanisms. The dominant eigenvalue of $\mathbf{G_0}$ is not simply deducible from the eigenvalues of these three addenda. Therefore, human mobility and hydrological networks interplay in a complex manner to determine parasite invasion and spread.

3 Spatial patterns of disease spread

Because of the assumption of strong connectivity made in the previous section, the condition under which the parasite can invade an initially disease-free community corresponds to that for the spatial spread of the disease. The spatial localization of the sites that are colonized by the parasite in the early phases following its introduction in a metacommunity is determined by the dominant eigenvector of the Jacobian matrix $\mathbf{J_0^*}$. In fact, if the DFE is unstable, the dominant eigenvector of matrix $\mathbf{J_0^*}$ pinpoints the directions in the state space along which the system trajectories, after a transient period related to initial conditions, will diverge from the equilibrium. The dominant eigenvector is characterized by strictly positive components [according to Perron-Frobenius theorem applied to Metzler matrices; see again **?**], each corresponding –

in this case – to the parasite burden in human hosts, the prevalence of infected snails, and the abundances of cercariae and miracidia in different nodes of the network.

The dominant eigenvector of $\mathbf{J_0^*}$ can be computed by solving

$$\mathbf{J_0^*}\begin{bmatrix}\mathbf{W}\\ \mathbf{Y}\\ \mathbf{C}\\ \mathbf{M}\end{bmatrix} = \lambda' \begin{bmatrix}\mathbf{W}\\ \mathbf{Y}\\ \mathbf{C}\\ \mathbf{M}\end{bmatrix},$$

where λ' is the dominant eigenvalue of $\mathbf{J_0^*}$, and $\mathbf{W}$, $\mathbf{Y}$, $\mathbf{C}$ and $\mathbf{M}$ are the components of the dominant eigenvector corresponding, respectively, to parasite burden, prevalence of infected snails, and abundances of cercariae and miracidia. Recalling that close to the transcritical bifurcation through which the DFE loses stability the dominant eigenvalue of $\mathbf{J_0^*}$ is equal to zero, the previous matrix equation becomes

$$\begin{aligned}
&-\gamma\mathbf{W} + a(\mathbf{I} - \mathbf{m} + \mathbf{mQ})\boldsymbol{\theta}\mathbf{C} = 0\\
&-\nu\mathbf{Y} + b\mathbf{M} = 0\\
&\Pi_C\mathbf{V}^{-1}\mathbf{NY} - (\mu_C\mathbf{I} - \mathbf{T_C})\mathbf{C} = 0\\
&\frac{\Pi_M}{2}\mathbf{V}^{-1}\boldsymbol{\theta}'(\mathbf{I} - \mathbf{m} + \mathbf{Q}^T\mathbf{m})\mathbf{HW} - (\mu_M\mathbf{I} - \mathbf{T_M})\mathbf{M} = 0\,.
\end{aligned}$$

From the first two equations we get

$$\mathbf{W} = \frac{a}{\gamma}(\mathbf{I} - \mathbf{m} + \mathbf{mQ})\boldsymbol{\theta}\mathbf{C}$$

and

$$\mathbf{Y} = \frac{b}{\nu}\mathbf{M}\,.$$

If we plug these two expressions into the third and fourth equations of the linear system above we find

$$\frac{b\Pi_C}{\nu}\mathbf{V}^{-1}\mathbf{NM} - (\mu_C\mathbf{I} - \mathbf{T_C})\mathbf{C} = 0$$

and

$$\frac{a\Pi_M}{2\gamma}\mathbf{V}^{-1}\boldsymbol{\theta}'(\mathbf{I} - \mathbf{m} + \mathbf{Q}^T\mathbf{m})\mathbf{H}(\mathbf{I} - \mathbf{m} + \mathbf{mQ})\boldsymbol{\theta}\mathbf{C} - (\mu_M\mathbf{I} - \mathbf{T_M})\mathbf{M} = 0\,.$$

Solving for $\mathbf{M}$ the first of these two equations

$$\mathbf{M} = \frac{\nu}{b\Pi_C}\mathbf{V}\mathbf{N}^{-1}(\mu_C\mathbf{I} - \mathbf{T_C})\mathbf{C}$$

and substituting into the second we get

$$\left[\frac{a\Pi_M}{2}\mathbf{V}^{-1}\boldsymbol{\theta}'(\mathbf{I} - \mathbf{m} + \mathbf{Q}^T\mathbf{m})\mathbf{H}(\mathbf{I} - \mathbf{m} + \mathbf{m}\mathbf{Q})\boldsymbol{\theta} - \frac{\gamma\nu}{b\Pi_C}(\mu_M\mathbf{I} - \mathbf{T_M})\mathbf{V}\mathbf{N}^{-1}(\mu_C\mathbf{I} - \mathbf{T_C})\right]\mathbf{C} = 0\ ,$$

which corresponds (see previous section) to

$$\mathbf{J_0^{***}}\mathbf{C} = 0\ .$$

Because we already know that

$$\mathbf{J_0^{***}} = \frac{\gamma\nu\mu_C\mu_M}{b\Pi_C}\mathbf{N}^{-1}\mathbf{J_0^{****}}\mathbf{V} = \frac{\gamma\nu\mu_C\mu_M}{b\Pi_C}\mathbf{N}^{-1}(\mathbf{G_0} - \mathbf{I})\mathbf{V}\ ,$$

combining the two previous expressions we have

$$(\mathbf{G_0} - \mathbf{I})\mathbf{C} = 0\ .$$

If we remember that close to the transcritical bifurcation of the DFE the dominant eigenvalue g_0 of matrix $\mathbf{G_0}$ is equal to one we can write

$$\mathbf{G_0}\mathbf{C} = \mathbf{C} = g_0\mathbf{C}\ .$$

We can thus conclude that close to the bifurcation through which the DFE loses stability the dominant eigenvector $\mathbf{g_0}$ of matrix $\mathbf{G_0}$ corresponds to the cercarial components of the dominant eigenvector of $\mathbf{J_0^*}$,

while the other components of $\mathbf{J_0^*}$ can be found as linear combinations of $\mathbf{g_0}$, i.e.

$$\mathbf{W} = \frac{a}{\gamma}(\mathbf{I} - \mathbf{m} + \mathbf{mQ})\boldsymbol{\theta}\mathbf{g_0}$$
$$\mathbf{Y} = \frac{1}{\Pi_C}\mathbf{VN}^{-1}(\mu_C\mathbf{I} - \mathbf{T_C})\mathbf{g_0}$$
$$\mathbf{C} = \mathbf{g_0}$$
$$\mathbf{M} = \frac{\nu}{b\Pi_C}\mathbf{VN}^{-1}(\mu_C\mathbf{I} - \mathbf{T_C})\mathbf{g_0}\ .$$

Note that these simple relationships between the dominant eigenvectors of $\mathbf{J_0^*}$ and $\mathbf{G_0}$ hold only close to the transcritical bifurcation of the DFE. In general, for parameter combinations for which $g_0 \gg 1$, the study of the geography of disease spread requires the computation of the eigenvalues and eigenvectors of matrix $\mathbf{J_0^*}$.
