# Supplementary material for: A Theoretical Analysis of the Geography of Schistosomiasis in Burkina Faso Highlights the Roles of Human Mobility and Water Resources Development in Disease Transmission
Source: PLoS Negl Trop Dis. 2015 Oct 29;9(10):e0004127. doi: 10.1371/journal.pntd.0004127 (PMC4625963; doi:10.1371/journal.pntd.0004127)
Supplement: S1 Table — Data sources, description and download links. (PDF) [file pntd.0004127.s002.pdf]

S1 Table: Spatial data sources, acknowledgements and access urls

Dataset	Figure	Description	Acknowledgements	Access url or ref.
Burkina Faso settlements	1(a)	Geo-location of villages and settlements in Burkina Faso	Second Administrative Level Boundaries (SALB)[i] project	`https://www.humanitarianresponse.info/en/operations/burkina-faso/dataset/burkina-faso-settlements-villages-towns-cities`
Road network	1(a)	Open Street Map road network data	Open Street Maps[ii] licensed under ODBL 1.0	`http://download.geofabrik.de/africa/burkina-faso.html`
Schistosomiasis prevalence	1(b)	Data consists of published parisotological studies in Burkina Faso from 1955 to 2007 (the average prevalence over survey years was taken in villages where multiple surveys were available)	Global Neglected Tropical Diseases Database	`www.gntd.org`
B.pfeifferi presence data	1(b)	Compilation of literature review data and sightings made during field campaigns from 1987 to 1992	-	Poda, 1996[iii]
River network	1(b)	River network based on HydroSHEDS flow accumulation raster	HydroSHEDS[iv]	`http://hydrosheds.cr.usgs.gov/dataavail.php`
Isohytes	1(b)	Decadal precipitation contours based on TRMM precipitation estimates	NASA Tropical Rainfall Measurement Mission Project (TRMM)[v]	`http://gcmd.nasa.gov/records/GCMD_GES_DISC_TRMM_3B42_daily_V6.html`
Base maps	1(c)	Open Street Maps base maps	Open Street Maps[vi] licensed under ODBL 1.0	`www.openstreetmap.org`
Population density	S1 Fig	Landscan 2011 population density product	Landscan product distributed by East View Information Services[vii]	can be replaced by WorldPop product[viii]

[i] `http://salbgeonetwork.grid.unep.ch/geonetwork/srv/en/main.home`
[ii] `www.openstreetmap.org/copyright`
[iii] Poda JN. Distribution spatiale des hôtes intermediaires des schistosomes au Burkina Faso: Facteurs infulen cant la dynamique des populations de Bulinus truncatus rohlfsi Classin, 1886 et de Bulinus sengalensis Muller, 1781 . Université de Ouagadougou; 1996.
[iv] Lehner, B., Verdin, K., Jarvis, A. (2008): New global hydrography derived from spaceborne elevation data. Eos, Transactions, AGU, 89(10): 93-94. ; Information: `http://www.worldwildlife.org/hydrosheds`
[v] Huffman, G.J., 1997: Estimates of Root-Mean-Square Random Error for Finite Samples of Estimated Precipitation, J. Appl. Meteor., 1191-1201.
[vi] `www.openstreetmap.org/copyright`
[vii] `www.eastview.com`
[viii] `http://www.worldpop.org.uk/`
