# Supplementary figures and images for: A Theoretical Analysis of the Geography of Schistosomiasis in Burkina Faso Highlights the Roles of Human Mobility and Water Resources Development in Disease Transmission
Source: PLoS Negl Trop Dis. 2015 Oct 29;9(10):e0004127. doi: 10.1371/journal.pntd.0004127 (PMC4625963; doi:10.1371/journal.pntd.0004127)

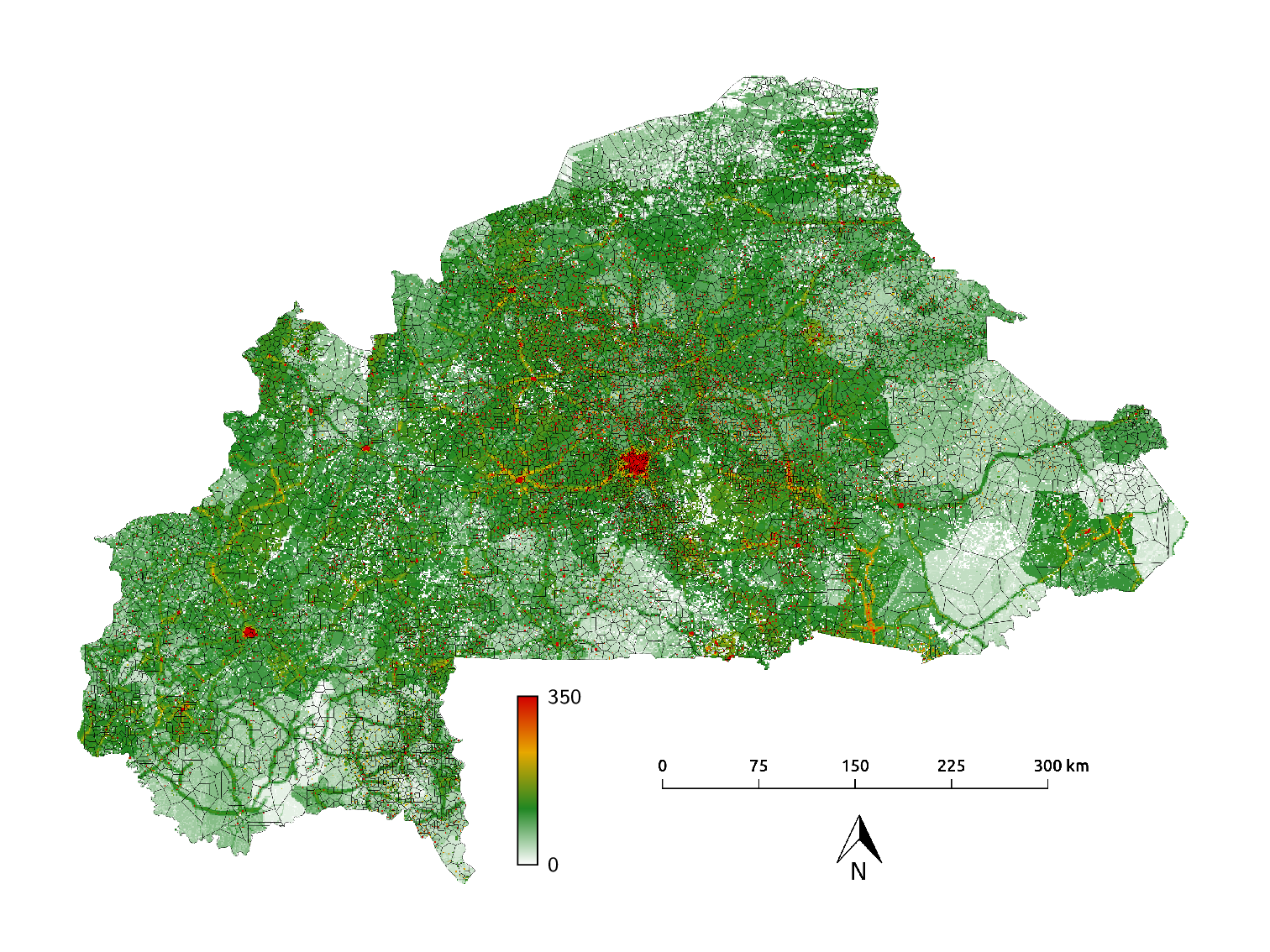

Supplement: S1 Fig — Population density is given at a resolution of ≈ 1km2. Permission to publish granted from East View Information Services/LandScan. (TIF) [file pntd.0004127.s003.tif]

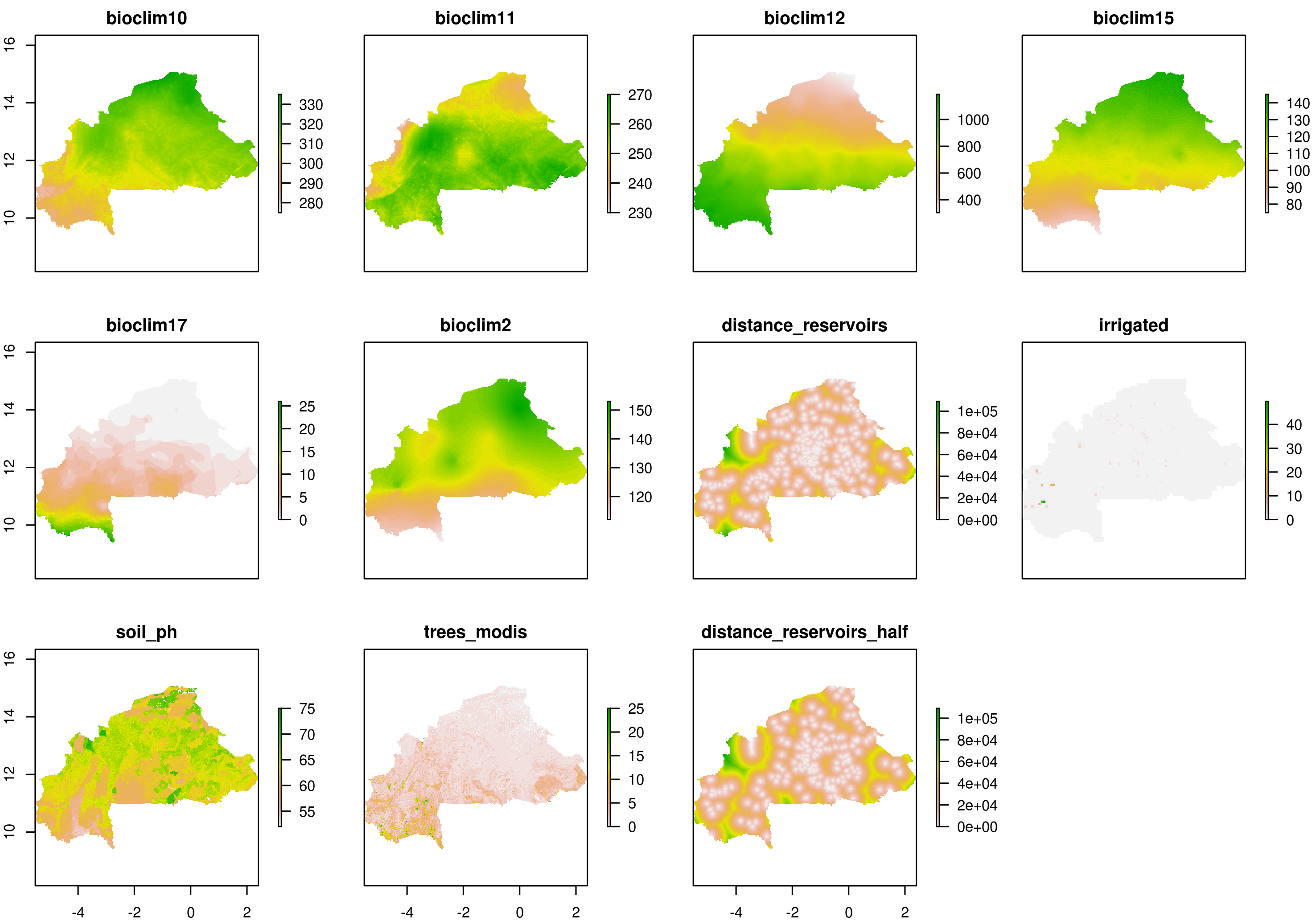

Supplement: S2 Fig — All data sources are analogous to those used in [72]. (TIF) [file pntd.0004127.s004.tif]

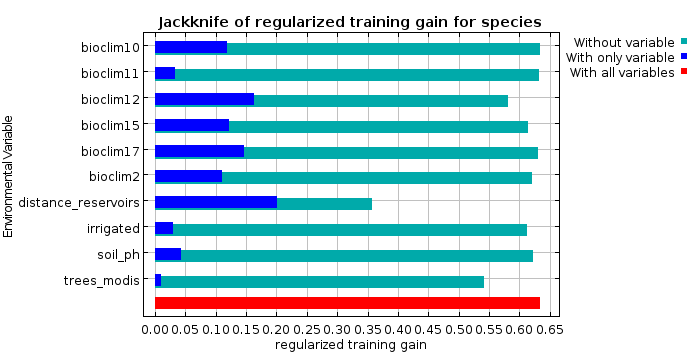

Supplement: S3 Fig — Results are shown for one fold of the fitting procedure. Importance is measured in terms of the regularized training gain of the MaxEnt model when removing a given variable (light blue) and using only that variable (dark blue). The former expresses the information contained in the given covariate that is different from other variables. The latter the specific explanatory power contained in the variable. The results show that distance to reservoirs is both the covariate with the largest explanatory power, and with the highest information content not contained in the other covariates. (TIF) [file pntd.0004127.s005.tif]

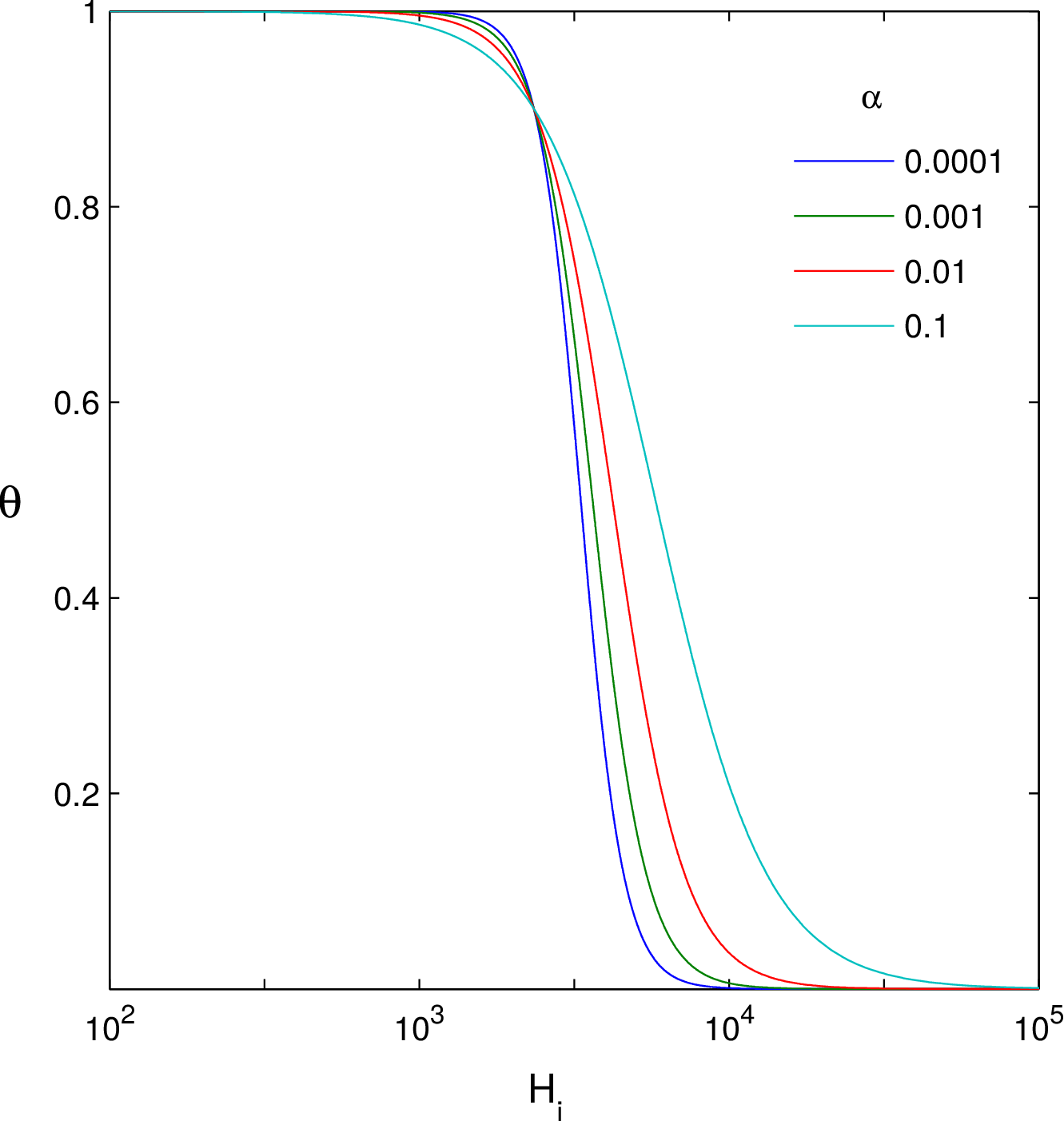

Supplement: S4 Fig — The curves are given here for θ MAX = 1 as a function of population in node i, H i, and for values of α used in model exploration. (TIF) [file pntd.0004127.s006.tif]

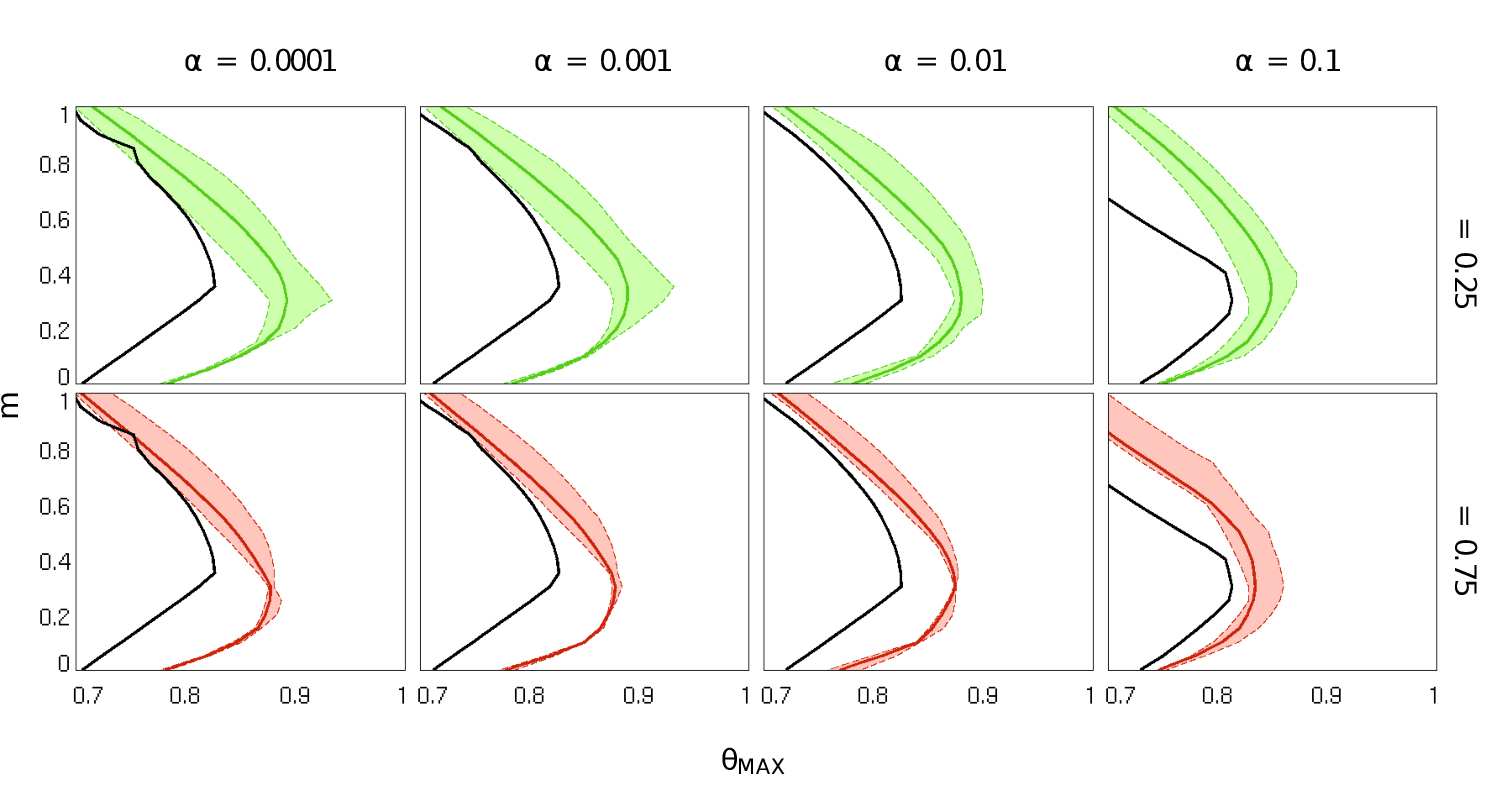

Supplement: S5 Fig — The bifurcation curves (g 0 = 1) of the DFE are plotted for different levels of water resources development expressed in terms of fraction of the existing reservoirs. Stability plots are given as a function of the maximal contact/exposure rate parameter θ MAX and the fraction of mobile people m. Coloured (green, red) curves represent parasite invasion conditions for the alternative scenarios of water resources development, while black lines refer to the current situation (same as Fig 2 in main text), and are reported here for reference. Colour shadings represent 95% confidence intervals based on 10 scenario realizations by random removal of existing reservoirs. Regions to the right of the black and coloured lines correspond to conditions of pathogen invasion of the country, i.e. an unstable DFE, for the current and alternative scenarios respectively. (TIF) [file pntd.0004127.s007.tif]
